# Tuning Matters: Comparing Lambda Optimization Approaches for Ridge Regression in Genomic Prediction

**DOI:** 10.3390/genes16060618

**Published:** 2025-05-23

**Authors:** Osval A. Montesinos-López, Eduardo A. Barajas-Ramirez, Abelardo Montesinos-López, Federico Lecumberry, María Inés Fariello, José Cricelio Montesinos-López, Juan Manuel Ramirez Alcaraz, José Crossa, Reka Howard

**Affiliations:** 1 Facultad de Telemática, Universidad de Colima, Colima 28040, Mexico; osval78t@gmail.com (O.A.M.-L.); ebarajas2@ucol.mx (E.A.B.-R.); jmramirez@ucol.mx (J.M.R.A.); 2 Centro Universitario de Ciencias Exactas e Ingenierías (CUCEI), Universidad de Guadalajara, Guadalajara 44430, Mexico; 3 Facultad de Ingeniería, Universidad de la República, Montevideo 11300, Uruguay; fefo@fing.edu.uy (F.L.); fariello@fing.edu.uy (M.I.F.); 4 Department of Public Health Sciences, University of California Davis, Davis, CA 95616, USA; jc.montesinos@gmail.com; 5 International Maize and Wheat Improvement Center (CIMMYT), Carretera Mexico-Veracruz, Km 45, Texcoco 52640, Mexico; j.crossa@cgiar.org; 6 Instituto de Estadística y Ciencias de los Datos (IECD), Colegio de Postgraduados, Texcoco 56230, Mexico; 7 Department of Statistics, 343C Hardin Hall Lincoln, University of Nebraska-Lincoln, Lincoln, NE 68583, USA

**Keywords:** ridge regression, prediction performance, tuning hyperparameter, continues response

## Abstract

Background/Objectives: Ridge regression (RR) is a widely used statistical learning method for predicting continuous response variables, particularly in high-dimensional contexts where the number of predictors (*p*) far exceeds the number of observations (*n*). RR is known for its simplicity, as it depends on a single regularization hyperparameter (λ), and for its strong predictive performance, especially in genomic prediction applications. However, selecting the optimal value of λ remains a key challenge, with standard techniques such as cross-validation often being computationally intensive and potentially suboptimal in terms of predictive accuracy. Methods: To address this issue, recent studies have proposed alternative methods for tuning λ, aiming to enhance both predictive power and computational efficiency. In this study, we perform a comprehensive benchmarking analysis of two novel λ-selection strategies and compare them with traditional approaches. The evaluation was conducted across 14 real-world genomic selection datasets, covering diverse scenarios representative of practical breeding programs. Results: Our results demonstrate that the method proposed consistently outperforms conventional approaches in both prediction accuracy and computational speed. Additionally, we found that combining this method with another recent approach yields a hybrid strategy that, in some cases, delivers the best overall performance. These findings underscore the importance of carefully selecting the regularization parameter in ridge regression models and suggest that modern, data-driven tuning approaches can substantially improve model performance. Conclusions: This study contributes valuable insights into optimizing hyperparameter selection for high-dimensional prediction problems, with direct implications for genomic selection and other applications in the life sciences.

## 1. Introduction

Ridge regression (RR), originally proposed by Hoerl and Kennard (1970) [1], is a fundamental method in statistical learning, widely used for predicting continuous outcomes in high-dimensional settings where the number of predictors (*p*) greatly exceeds the number of observations (*n*). This scenario is common in genomic prediction, where tens of thousands of molecular markers are used to predict phenotypes in relatively small populations. RR addresses multicollinearity and overfitting by adding a penalty to the size of the regression coefficients, effectively shrinking them toward zero. The amount of shrinkage is governed by a single regularization hyperparameter, which must be carefully chosen to balance the trade-off between model complexity and predictive accuracy.

Traditionally, λ is selected using data-resampling methods such as k-fold cross-validation (CV), leave-one-out CV, and generalized cross-validation (GCV) (Golub et al., 1979) [2]. These methods aim to minimize prediction errors on held-out data but can be computationally intensive, especially in genomic contexts involving multiple traits and models. Furthermore, their performance may be unstable in the presence of noisy or unbalanced data.

To address these limitations, model-based alternatives have been proposed. One popular approach is based on restricted maximum likelihood (REML) estimation, especially when RR is reparametrized as a linear mixed model (LMM). In this formulation, λ corresponds to the ratio of error variance to genetic variance. This connection was exploited by de los Campos et al. (2009) [3], who developed a framework for best genomic linear unbiased prediction (GBLUP) under LMMs, effectively embedding RR within a broader quantitative genetics’ context. REML-based tuning offers statistical rigor but may also be computationally demanding in large-scale genomic applications.

Another class of approaches involves empirical Bayes estimation, where λ is estimated by maximizing the marginal likelihood of the data (Zhang and Huang, 2008) [4]. This approach is particularly appealing when prior distributions for regression coefficients are assumed, as it provides shrinkage estimates with closed-form or iterative solutions. Akdemir and Okeke (2015) [5] further contributed to this area by proposing heuristic algorithms for tuning shrinkage parameters in multi-environment genomic prediction models, showing improved prediction in plant breeding scenarios.

A complementary yet distinct approach is offered by predictive scoring rules, as introduced by Czado, Gneiting, and Held (2009) [6]. This framework evaluates the quality of predictive distributions using strictly proper scoring rules, such as the logarithmic score and Brier score, which reward accurate and well-calibrated probabilistic forecasts. A scoring rule is strictly proper if it encourages honest predictions by assigning the best expected score when the forecast matches the true distribution. The logarithmic score penalizes predictions that assign low probability to observed outcomes, while the Brier score measures the mean squared difference between predicted probabilities and actual outcomes. Though initially developed for discrete data, these rules extend to continuous settings. By capturing both calibration (alignment with observed frequencies) and sharpness (concentration of predictions), scoring rules offer a statistically principled way to compare models and assess the effect of different λ values—without relying solely on resampling or point estimates.

Pavlou et al. (2024) [7] address the challenge of overfitting in risk prediction models, particularly when using penalized regression techniques like Ridge and Lasso. Traditional cross-validation (CV) methods for selecting the tuning parameter (λ) often lead to over-shrinkage, especially in small sample sizes, resulting in predictions that are too conservative and calibration slopes (CS) less than 1. To mitigate this, the authors propose a modified cross-validation tuning method, which involves: (1) generating a pseudo-development dataset by bootstrapping from the original dataset. This pseudo-dataset is larger, ensuring that the subsequent training datasets used in CV are of the same size as the original dataset; (2) applying cross-validation on these bootstrapped datasets, which helps in more accurately estimating the optimal λ by maintaining the sample size consistency; and (3) exploring a bootstrap-based method for tuning λ, which involves repeatedly resampling the data and selecting λ that minimizes prediction error across these samples. These approaches aim to provide better estimates of λ, leading to improved model calibration and prediction accuracy.

Building on these innovations, Montesinos-López et al. (2024) [8] recently introduced a Bayesian asymmetric loss framework for selecting λ, specifically developed for genomic prediction. Their method defines a loss function that differentially penalizes overestimation and underestimation, aligning model optimization with biological or economic priorities in breeding programs. Rather than relying on repeated cross-validation, they derive an analytical expression for λ that minimizes the expected asymmetric loss using the posterior predictive distribution. This results in a more adaptive and computationally efficient estimation of the shrinkage parameter.

Importantly, Montesinos-López et al. (2024) [8] also explored a hybrid strategy that combines their asymmetric loss criterion with another recent optimization framework, demonstrating superior performance in terms of both accuracy and execution time across multiple genomic datasets.

Despite the growing number of lambda selection methods, there is still a lack of systematic comparisons across a broad set of real-world genomic datasets, especially in the context of high-dimensional, small-sample settings commonly found in plant and animal breeding. Many existing studies focus on isolated methods or use simulated data, which limits generalizability to practical breeding applications. This study addresses this critical gap by performing a comprehensive benchmarking of recent lambda selection strategies, including biologically motivated approaches and hybrid methods. The novelty of this work lies in its rigorous empirical comparison across 14 diverse datasets, offering practical insights into the trade-offs between prediction accuracy and computational cost. Our contribution is two-fold: (1) we validate the effectiveness of the Montesinos-López et al. method in improving predictive accuracy and computational efficiency, and (2) we demonstrate that hybrid methods can further optimize model performance in complex breeding scenarios.

In this study, we perform a systematic comparison of the six most prominent recent strategies for selecting λ in ridge regression. We evaluate their predictive performance and runtime using 14 publicly available genomic selection datasets. Our results offer new insights into the practical trade-offs between flexibility, statistical rigor, and computational cost in shrinkage-based genomic prediction models.

## 2. Material and Methods

### 2.1. Data Availability

The data used in this study is available at https://github.com/osval78/Refaning_Penalized_Regression (accessed on 1 January 2024).

Table 1 outlines the general information of the 14 datasets used in this study. These datasets are available at: https://github.com/osval78/Refaning_Penalized_Regression (accessed on 1 January 2024).

### 2.2. The Ridge Regression Methods

Tuning the λ (lambda) parameter in ridge regression is essential because it controls the amount of shrinkage applied to the model coefficients. This helps to balance the bias-variance tradeoff, reducing overfitting in high-dimensional genomic data. An appropriately chosen λ improves prediction accuracy and model robustness, especially when the number of markers exceeds the number of observations.

The strategies used in this study are divided into two groups: (1) the conventional (standard) Ridge Regression (RR), using traditional λ tuning techniques, and (2) a set of improved or novel methods (RRE, RRBE, ML, MRG, MRG-ML, MRGE) that enhance model performance by adopting more refined and adaptive λ selection procedures.

#### 2.2.1. Standard Ridge Regression (RR)

Within a general framework, let $x_{i}={\left(x_{i1},\;\ldots ,\;x_{ip}\right)}^{T},$ i=1, …, n, denote a vector of covariates. The goal is to utilize this information to predict or interpret the influence of these variables on a continuous outcome $y_{i}$. The multiple linear regression model establishes a relationship between the covariate vectors and the response variable as follows:

(1) $$y_{i}=\beta_{0}+\sum_{j=1}^{p}x_{ij}\beta_{j}+\epsilon_{i}$$

In this context, $\epsilon_{i}\;$ denotes a random error term with expected value zero, $E\left(\epsilon_{i}\right)=0$, and is assumed to be independent of $x_{i}$. This term captures the effects of measurement inaccuracies and the impact of unobserved factors that may also influence the response variable. The conditional expectation of the response, given the covariates, is expressed as $E\left(y_{i}\;|x_{i}\right)=\beta_{0}+\sum_{j=1}^{p}x_{ij}\beta_{j}.\;$ Thus, the conditional distribution of $y_{i}$, given $x_{i}$ is entirely determined by the information contained in the covariate vector $x_{i}$.

To estimate the parameter vector $\beta ={\left(\beta_{0},\beta_{1},\;\ldots ,\;\beta_{p}\right)}^{T}$, we typically utilize a dataset $\left(x_{i}^{T},y_{i}\right)$, i=1, …, n, commonly referred to as the training set. In this dataset, $x_{i}\;$denotes the vector of predictor values, while $y_{i}\;$ is the observed outcome for the i-th subject. In scenarios where the number of predictors p  is large relative to the sample size n, RR is among the most frequently applied techniques for estimating β. This approach seeks the values of β that minimize the penalized residual sum of squares, as described in Montesinos-López et al. (2022) [9], and is defined as follows:

(2) $$PRSS_{\lambda }\left(\beta \right)={\left(y-X\beta \right)}^{T}(y-X\beta )+\lambda \sum_{j=1}^{p}\beta_{j}^{2}$$

In this context, λ≥0  denotes the regularization parameter, which governs the degree to which the regression coefficients are shrunk toward zero. When λ = 0, the resulting estimates coincide with those obtained from the ordinary least squares (OLS) method. As λ increases, however, the penalized residual sum of squares $PRSS_{\lambda }\left(\beta \right),\;$becomes increasingly influenced by the penalty term, which progressively shrinks the OLS estimates toward zero (Christensen, 2011) [10]. This regularization is particularly beneficial in situations where the number of predictors exceeds the number of observations, a setting in which the OLS estimator is no longer viable. RR addresses this challenge by introducing a constraint on the sum of squares of the regression coefficients, thereby stabilizing the estimation procedure (Wakefield et al., 2013) [11]. When the optimization of the objective function in Equation (2) is performed using the glmnet library (Friedman, Hastie, & Tibshirani, 2010) [12], the resulting approach is referred to as the standard ridge RR. In RR, the value of λ is typically selected through cross-validation on the training data. This is commonly accomplished using the default grid search procedure implemented in the cv.glmnet function of the glmnet R package (Friedman et al., 2010) [12].

In this study, the standard cross-validation method of RR is as follows:

Step 1: The original development dataset of size n is randomly divided into k subsets. Common choices for k include 5 or 10.

Step 2: A cross-validation training set (CV-training set) is created by combining k−1 of these subsets, while the remaining subset is used as a cross-validation test set (cv-test set). A range of values for the regularization parameter λ is selected, and for each value, the model is trained on the CV-training set using the chosen penalized regression method.

Step 3: This process is repeated k times, each time leaving out a different subset for testing.

Step 4: For every value of λ, an out-of-sample performance metric—such as deviance, C-statistic, or mean absolute error—is computed using the corresponding cv-test sets. In this study, deviance is used as the performance criterion.

Step 5: The optimal value λ, λmin, is identified as the one that results in the lowest cross-validated deviance.

Step 6: The regression coefficients are then estimated by fitting the RR model on the full original development dataset, using λ = λmin.

#### 2.2.2. Ridge Regression Extended (RRE)

This method is a slight yet purposeful modification of standard RR. While it retains the core structure of RR, the key distinction lies in the selection of the tuning parameter λ. Instead of relying on the default grid of 100 λ values—commonly employed in standard implementations such as the *glmnet* package—this approach expands the grid to include 119 values. Specifically, it incorporates the original 100 values along with an additional set of 19 values concentrated at the lower end of the λ spectrum, thereby extending the sequence.

These additional values are constructed by taking the smallest value in the original grid, denoted as $\lambda_{ngrid}$, and generating a new sequence defined as ${\left\{\lambda_{ngrid}\left(1-\frac{n}{20}\right)\right\}}_{n=0}^{19}.$ This results in an equally spaced grid ranging from ${\;\lambda }_{ngrid}$ to $\frac{\lambda_{ngrid}\;}{20}$, providing finer granularity in the region of small λ values, where model performance can be particularly sensitive. This extension strategy underlies all the methods labeled with the term extended in this paper, reflecting their shared enhancement to the standard tuning grid.

#### 2.2.3. Ridge Regression with Bootstrap Extended (RRBE)

In this method we apply the standard bootstrap resampling approach to select the training and validation sets internally. After that, in the outer training set, we apply the RRE method to select the optimal λ. This gives us an out-of-sample performance measure, which in our case is the average MSE. Then, the bootstrap resampling is iterated BS=100 times, and the model with the optimal out-of-sample performance metric is selected. Bootstrap consists of selecting the rows of the training set randomly and with replacement. This leads to the duplication of some rows and some unchosen rows; the latter rows will form the validation set. In this approach, for each candidate value of λ, the model is trained on a bootstrap sample, and its out-of-sample performance is evaluated using the original dataset. We refer to this procedure as “bootstrap tuning”. The optimal value of λ is identified as the one that maximizes the average performance—such as deviance—across multiple bootstrap iterations.

#### 2.2.4. Menelao-Rumana-Gareth Ridge Regression Model (MRG)

This method uses Ridge Regression with the cross-validation technique detailed in Pavlou et al. (2024) [7] specified as Modified Tuning Cross-Validation. This method, denoted as MRG, is closely related to Bootstrap tuning and was proposed with the intention of improving the selection of λ in the cross-validation process. For our purposes, the number of bootstrap samples was B=100  as suggested in the paper of Pavlou et al. (2024) [7], and we considered the mean square error (MSE) as the measure of out-of-sample performance. The detailed steps for this method are provided as follows:

**Step 0:** Generate a pseudo-sample from the original development dataset by sampling with replacement, resulting in a dataset of size $n_{pseudo}=n\frac{k}{k-1}$, which exceeds the size of the original data.

**Steps 1–4:** These steps follow the same procedure as in the standard tuning algorithm of RR but are applied to the pseudo-development dataset generated in Step 0. A key feature of this approach is that the resulting CV-training sets obtained during k-fold cross-validation are equal in size to the original dataset.

**Step 5:** Repeat Steps 0 through 4 a total of B times, and select the value of λ, denoted as λmod, that minimizes the average cross-validation across all B repetitions. Alternatively, one may compute λmin within each pseudo-dataset and then select the median of these values as λmod, which yields practically equivalent outcomes.

**Step 6:** Using the selected penalized estimation method and the optimal tuning parameter λmod, fit the model to the original development dataset.

#### 2.2.5. Montesinos-López et al. [8,9] Method (ML)

This is the method given in Montesinos-López et al. (2024) [8]. This method is inspired by a mixed (or Bayesian) model framework, where λ is estimated as a ratio of variance components as ${\lambda =\sigma }^{2}/\sigma_{\beta }^{2}$, where $\sigma^{2}$ is the variance of the error term and $\sigma_{\beta }^{2}$ is the variance of the beta coefficients, which guarantees a lower mean squared error (MSE) in future predicted values (Montesinos-López et al., 2022) [9]. The customized grid values of λ under this method are computed as

(3) $$\lambda_{l}=\frac{\sigma_{l}^{2}}{\sigma_{l\beta }^{2}}=\frac{\left(1-R_{l}^{2}\right)s_{y}^{2}}{R_{l}^{2}s_{y}^{2}/\left(\frac{1}{n_{trn}}\sum_{i=1}^{n_{trn}}x_{i}^{T}x_{i}\right)}=\frac{1-R_{l}^{2}}{R_{l}^{2}/\left(\frac{1}{n_{trn}}\sum_{i=1}^{n_{trn}}x_{i}^{T}x_{i}\right)},\;l=1,\ldots ,100\;$$

where $R_{l}^{2}$ denotes a proportion of the genotypic variance explained by the $x_{i}^{T}\beta$ term (genotypic effects), starting from a small value ($10^{-5}$) up to a large value (0.999). The term $s_{y}^{2}$ represents the phenotypic variance in the training data, and

(4) $$\sigma_{l\beta }^{2}=\frac{R_{l}^{2}s_{y}^{2}}{\left(\frac{1}{n_{trn}}\sum_{i=1}^{n_{trn}}x_{i}^{T}x_{i}\right)}$$

and $\sigma_{l}^{2}=\left(1-R_{l}^{2}\right)s_{y}^{2}$ represents the remaining proportion of the phenotypic variance left to the error variance, where the computation of $R_{l}^{2}$ is based on Montesinos-López et al. (2024) [8] as

(5) $$R_{l}^{2}=exp\left(lR_{l}\right)$$

and

(6) $$lR_{l}=log\;\left(10^{-5}\right)+[\frac{\left[log\;\left(0.9999\right)-log\;\left(10^{-5}\right)\;\right]}{99}]\times (l-1),\;$$

where l=1,…,100, are the different proportions of phenotypic variance are explained by the genotypic effects to be explored. For each value of λ in this grid, the average performance prediction, measured by the mean square error (MSE computed as $\sum_{i=1}^{n_{val}}{\left(y_{i}-\hat{y_{i}}\right)}^{2}$ where $n_{val}$ denotes the number of observations in the validation set and $\hat{y_{i}}$ denotes the predicted value i), was obtained across an inner 10-fold cross-validation strategy. Then, the value of λ that corresponds to the smallest MSE in this grid in the validation data is chosen as the optimal λ value. Subsequently, the model is fitted with the entire training set using this optimal value, which is then evaluated on the testing set. More details of this method can be found in Montesinos-López et al. (2024) [8].

#### 2.2.6. Joining MRG-ML and MRGE Methods

The MRG-ML method consists of selecting the values of λ as proposed by the ML method and the cross-validation tuning proposed in the MRG approach described by Pavlou et al. (2024) [7]. This hybrid method (MRG plus ML methods) is denoted as the **MRG-ML** method. On the other hand, the **MRGE** method consists of the extended technique plus the MRG approach for cross-validation.

Section 2.2.1, Section 2.2.2, Section 2.2.3, Section 2.2.4, Section 2.2.5 and Section 2.2.6 contain the description of the methods with specific explanation of the terms. However, the more frequently used symbols are not defined in each section. To ensure that the terms that appear in most of these sections are explained and the equation that defines the term is included (if applicable), we created a nomenclature table. Also, we synthesized the lambda-tuning methods and compared them in terms of their advantages and some of their characteristics. The nomenclature table with equation and the comparison of lambda tuning strategies in Ridge Regression are given below in Table 2 and Table 3, respectively.

#### 2.2.7. Evaluation Metrics

To assess model performance, we used the following evaluation metrics:

1. Pearson’s Correlation (Cor): (Cor = cov(y, ŷ)/(σ_γ_ σ_ŷ_) Measures the linear association between the predicted and observed phenotypic values across folds. Higher values indicate better predictive accuracy.
2. Normalized Root Mean Square Error (NRMSE): This is a scale-independent measure of prediction error, calculated as NRMSE = √[Σ(y_i_ − ŷ_i_)^2^/n]/ȳ where y_i_ and ŷ_i_ represent the observed and predicted values, respectively, n is the number of test samples, and ȳ is the mean of the observed values in the validation set.

Normalization Note: We applied the most common normalization approach, dividing the RMSE by the mean of the observed response variable ȳ. This ensures comparability across traits with different scales.

3. Standard Deviations of Normalized Root Mean Square Error and Pearson’s Correlation (NRMSE_SD and Cor_SD): Computed across the k-folds to quantify variability in the metrics due to data partitioning. These values give insight into the stability of each method.
4. Execution Time (Time): Measured in seconds, indicating the average computational time taken by each method to complete training and tuning on a dataset.
5. Standard Deviation of Execution Time (Time_SD): Denotes the standard deviation of execution time across k-folds, reflecting the consistency of computational performance.

#### 2.2.8. Cross Validation Strategy

Externally, a k-fold cross-validation (CV) strategy was used for all the methods evaluated (with k=10) while internally the cross-validation depended on the method. In the RR and RRE methods, the CV strategy is the standard one used in glmnet. In the ML method, a stratified k-fold cross-validation was implemented (with k=10). RRBE uses bootstrap resampling plus the standard cross-validation strategy of glmnet. The MRG and MRGE methods use a modified version of bootstrap tuning, which is described in more detail later, and the standard glmnet CV. Finally, the MRG-ML method uses stratified k-fold cross-validation (with k=10) plus the modified version of bootstrap tuning.

## 3. Results

In this section we highlight the results of 4 of the 14 datasets used in this study (Disease, EYT_1, Groundnut, and Japonica) and portrayed in Figure 1, Figure 2, Figure 3 and Figure 4 and Table 4, Table 5, Table 6, Table 7 and Table 8. Also, we present the average results across all datasets in Figure 5 and Table 9. The results of the remaining datasets (EYT_2, EYT_3, Indica, Maize, Wheat_1, Wheat_2, Wheat_3, Wheat_4, Wheat_5, and Wheat_6) and the time execution benchmarking figures are provided in Supplemental Materials, which contains Tables S1–S10 and Figures S1–S25. In each subsection, we compare the performance of the best method (considering Pearson’s correlation) with the performance of the remaining methods in terms of Cor, NRMSE, and time of execution. The methods compared are RR, RRE, RRBE, MRG, MRGE, ML, and MRG-ML.

### 3.1. Dataset Disease

**Figure 1:**
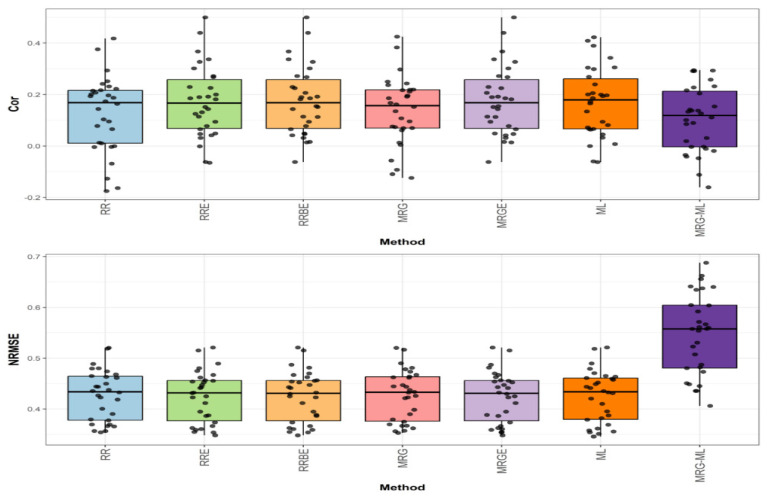
Box plots of Pearson’s correlation (Cor) and normalized root mean square error (NRMSE) for the “Disease” dataset are presented in the top and bottom graphs respectively. Each black dot represents a data point per fold and trait.

Figure 1 shows the prediction performance in terms of NRMSE and Cor for the Disease dataset.

In the first comparison for disease, which can be found in the top graph of Figure 1, we can observe that, in terms of Cor, the RRBE and MRGE methods, with an average correlation value of 0.176, proved to be slightly superior to the RRE and ML methods (by 2.326% and 4.756%, respectively), which exhibited average correlation values of 0.172 and 0.168, respectively. The RRBE and MRGE methods were also significantly superior to the MRG, RR, and MRG-ML methods (by 29.412%, 40.800%, and 67.619%) when they showcased an average correlation value of 0.136, 0.125, and 0.105, respectively. The individual values can be observed in more detail in Table 4.

**Table 4:** Prediction performance in terms of normalized root mean square error (NRMSE), Pearson’s correlation (Cor), and average execution of model (Time) for the Disease dataset. NRMSE_SD denotes the standard deviation of NRMSE, Cor_SD denotes the standard deviation of Cor, and Time_SD denotes the standard deviation of Time.

Dataset	Method	Cor	Cor_SD	NRMSE	NRMSE_SD	Time	Time_SD
Disease	RR	0.125	0.147	0.427	0.050	0.739	0.050
Disease	RRE	0.172	0.138	0.424	0.050	0.739	0.050
Disease	RRBE	0.176	0.134	0.424	0.050	84.199	3.067
Disease	MRG	0.136	0.133	0.427	0.050	74.083	1.748
Disease	MRGE	0.176	0.134	0.424	0.050	84.341	3.733
Disease	ML	0.168	0.135	0.425	0.051	0.900	0.055
Disease	MRG-ML	0.105	0.128	0.547	0.079	93.682	3.83

In the second comparison for Disease, found in the bottom graph of Figure 1, we note that in terms of NRMSE, the methods ML, RR, MRG, and MRG-ML reported corresponding average NRMSE values of 0.425, 0.427, 0.427, and 0.547, while the methods that used the extended technique (RRE, RRBE, MRGE) reported an average NRMSE value of 0.424, being superior by 0.236% to the ML method, by 0.708% to the RR and MRG methods, and by 29.009% to the MRG-ML method. Detailed individual values can be observed in Table 4.

In the third and final comparison (Supplementary Materials Figure S1), we can observe the time of execution of the different methods, and we appreciate that the RR and RRE were the fastest, with an average time of 0.739 s.; then we have the ML method, which is slightly slower, with a time of 0.9 s.; and finally, we have that the MRG, RRBE, MRGE, and MRG-ML methods were significantly slower, with an average execution time of 74.083 s., 84.199 s., 84.341 s., and 93.682 s., respectively. For more details, consult Table 4.

### 3.2. Dataset EYT_1

**Figure 2:**
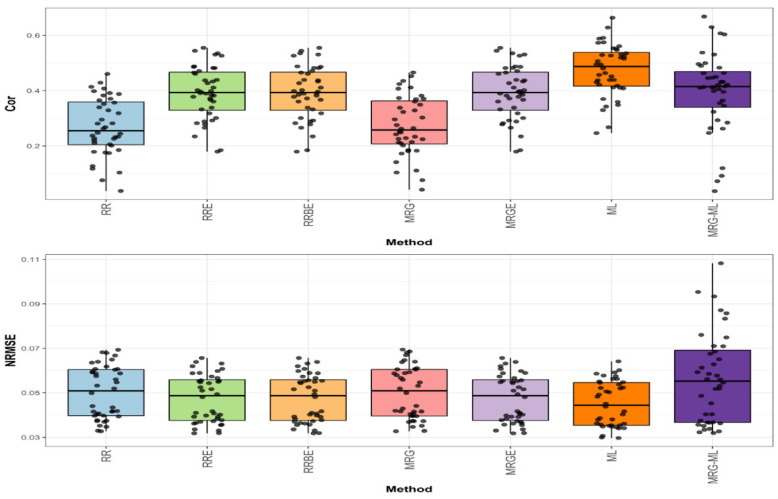
Box plots of Pearson’s correlation (Cor) and normalized root mean square error (NRMSE) for the “EYT_1” dataset are presented in the top and bottom graphs, respectively. Each black dot represents a data point per fold and trait.

Figure 2 presents the prediction performance for the EYT_1 dataset in terms of NRMSE and Cor.

In the first comparison (top graph of Figure 2), the highest correlation was achieved by the ML method, with an average correlation value of 0.474. This method outperformed the RRE, RRBE, and MRGE methods by 20.918%, as these methods obtained an average correlation value of 0.392. The ML method also showed a notable improvement over the MRG-ML method (by 19.697%), the MRG method (by 74.265%), and the RR method (by 76.208%), which exhibited average correlation values of 0.396, 0.272, and 0.269, respectively. The individual values can be observed in Table 5.

**Table 5:** Prediction performance in terms of normalized root mean square error (NRMSE), Pearson’s correlation (Cor), and average execution of model (Time) for the EYT_1 dataset. NRMSE_SD denotes the standard deviation of NRMSE, Cor_SD denotes the standard deviation of Cor, and Time_SD denotes the standard deviation of Time.

Dataset	Method	Cor	Cor_SD	NRMSE	NRMSE_SD	Time	Time_SD
EYT_1	RR	0.269	0.104	0.050	0.012	1.030	0.058
EYT_1	RRE	0.392	0.096	0.047	0.011	1.030	0.058
EYT_1	RRBE	0.392	0.096	0.047	0.011	122.434	2.519
EYT_1	MRG	0.272	0.107	0.050	0.012	112.056	1.695
EYT_1	MRGE	0.392	0.096	0.047	0.011	134.871	2.129
EYT_1	ML	0.474	0.095	0.045	0.01	1.366	0.129
EYT_1	MRG-ML	0.396	0.144	0.057	0.02	160.022	13.541

In the second comparison (bottom graph of Figure 2), regarding NRMSE, the ML method achieved the lowest average NRMSE value of 0.045, serving as the baseline. The RRE, RRBE, and MRGE methods presented a slightly higher average NRMSE value of 0.047, reflecting an increase of 4.444%. Meanwhile, the RR and MRG methods reported an average NRMSE value of 0.050, representing an 11.111% increase. The MRG-ML method exhibited the highest NRMSE at 0.057, marking a 26.667% increase. Detailed individual values are available in Table 5.

Finally, in the execution time comparison (Figure S2), the RR and RRE methods were the fastest, with an average execution time of 1.03 s. The ML method followed with a slightly higher time of 1.366 s. In contrast, the MRG, RRBE, MRGE, and MRG-ML methods were significantly slower, with average execution times of 112.056 s., 122.434 s., 134.871 s., and 160.022 s., respectively. Further details can be found in Table 5.

### 3.3. Dataset Groundnut

**Figure 3:**
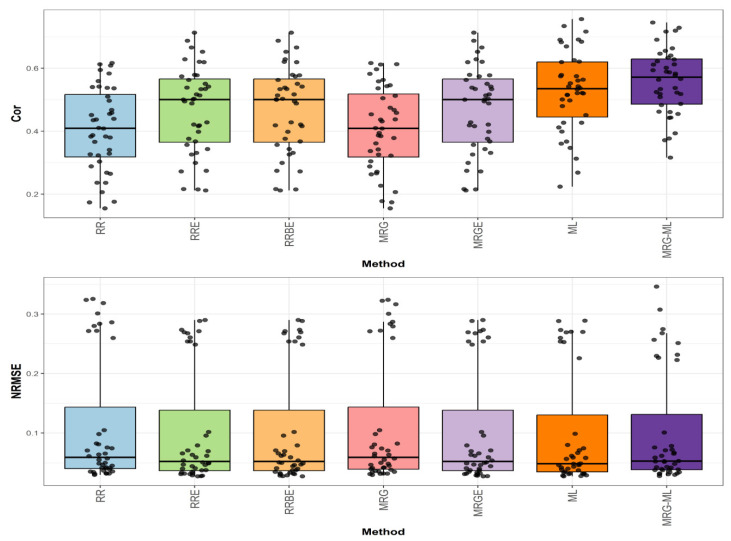
Box plots of Pearson’s correlation (Cor) and normalized root mean square error (NRMSE) for the “Groundnut” dataset are presented in the top and bottom graphs, respectively. Each black dot represents a data point per fold and trait.

Figure 3 illustrates the prediction performance for the Groundnut dataset in terms of NRMSE and Cor.

In the first comparison for Groundnut (Figure 3), the highest correlation was achieved by the ML method, with an average correlation value of 0.641. This method demonstrated an advantage over the RRE, RRBE, and MRGE methods by 55.206%, as these methods reached an average correlation value of 0.413. Similarly, the ML method surpassed the MRG-ML method by 2.889%, while the MRG and RR methods displayed the lowest average correlation values at 0.34 and 0.338, trailing behind the ML method by 88.529% and 89.645%, respectively. The individual values are detailed in Table 6.

In the second comparison (Figure 3), concerning NRMSE, the ML method reported the lowest average NRMSE value of 0.217, serving as the reference. The RRE, RRBE, and MRGE methods exhibited a higher average NRMSE value of 0.263, reflecting a difference of 21.198%. The MRG and RR methods showed slightly higher values at 0.272 and 0.273, representing differences of 25.346% and 25.806%, respectively. The MRG-ML method, with an average NRMSE value of 0.224, had a smaller difference of 3.226% compared to the ML method. Further details are provided in Table 6.

In the execution time comparison (Supplementary Materials Figure S3), the RR and RRE methods displayed the shortest execution times, averaging 0.453 s. The ML method required a slightly longer duration at 0.803 s. Conversely, the MRG, RRBE, MRGE, and MRG-ML methods took significantly more time, with execution times of 45.524 s., 52.33 s., 54.059 s., and 86.323 s., respectively. Detailed values can be reviewed in Table 6.

**Table 6:** Prediction performance in terms of normalized root mean square error (NRMSE), Pearson’s correlation (Cor), and average execution of model (Time) for the Groundnut dataset. NRMSE_SD denotes the standard deviation of NRMSE, Cor_SD denotes the standard deviation of Cor, and Time_SD denotes the standard deviation of Time.

Dataset	Method	Cor	Cor_SD	NRMSE	NRMSE_SD	Time	Time_SD
Groundnut	RR	0.338	0.211	0.273	0.051	0.453	0.036
Groundnut	RRE	0.413	0.198	0.263	0.049	0.453	0.036
Groundnut	RRBE	0.413	0.198	0.263	0.049	52.330	0.958
Groundnut	MRG	0.340	0.211	0.272	0.051	45.524	1.165
Groundnut	MRGE	0.413	0.198	0.263	0.049	54.059	0.857
Groundnut	ML	0.641	0.118	0.217	0.040	0.803	0.178
Groundnut	MRG-ML	0.623	0.133	0.224	0.048	86.323	20.647

### 3.4. Dataset Japonica

**Figure 4:**
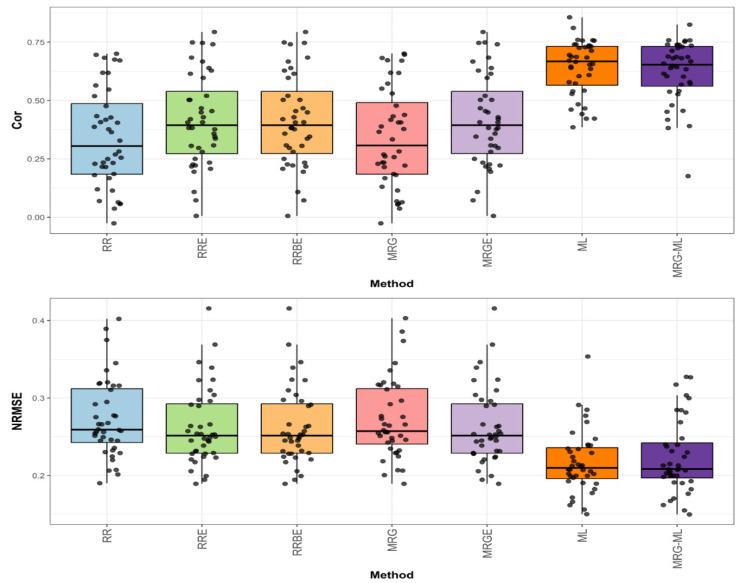
Box plots of Pearson’s correlation (Cor) and normalized root mean square error (NRMSE) for the “Japonica” dataset are presented in the top and bottom graphs, respectively. Each black dot represents a data point per fold and trait.

Figure 4 presents the prediction performance for the Japonica dataset in terms of NRMSE and Cor.

The first comparison (Figure 4) shows that the MRG-ML method achieved the highest correlation, with an average correlation value of 0.558, serving as the reference. The ML method followed closely with a correlation value of 0.528, trailing by 5.682%. The RRE, RRBE, and MRGE methods exhibited a correlation value of 0.469, reflecting an 18.977% decrease compared to MRG-ML. Meanwhile, the MRG and RR methods performed notably worse, with correlation values of 0.408 and 0.406, lagging behind by 36.765% and 37.438%, respectively. Detailed values are available in Table 7.

**Table 7:** Prediction performance in terms of normalized root mean square error (NRMSE), Pearson’s correlation (Cor), and average execution of model (Time) for the Japonica dataset. NRMSE_SD denotes the standard deviation of NRMSE, Cor_SD denotes the standard deviation of Cor, and Time_SD denotes the standard deviation of Time.

Dataset	Method	Cor	Cor_SD	NRMSE	NRMSE_SD	Time	Time_SD
Japonica	RR	0.406	0.132	0.113	0.107	0.451	0.053
Japonica	RRE	0.469	0.137	0.104	0.098	0.451	0.053
Japonica	RRBE	0.469	0.137	0.104	0.098	51.594	0.864
Japonica	MRG	0.408	0.133	0.112	0.107	44.080	0.783
Japonica	MRGE	0.469	0.137	0.104	0.098	51.696	0.839
Japonica	ML	0.528	0.128	0.101	0.097	0.550	0.169
Japonica	MRG-ML	0.558	0.104	0.102	0.096	62.824	20.898

For the second comparison for Japonica, found in Figure 4, the ML method achieved the lowest average NRMSE value of 0.101, serving as the baseline. The MRG-ML method was marginally worse with an average NRMSE value of 0.102, with a 0.99% increase. The RRE, RRBE, and MRGE methods reported an average NRMSE value of 0.104, reflecting a 2.97% increase. The MRG and RR methods performed the worst, with NRMSE values of 0.112 and 0.113, representing increases of 10.891% and 11.881%, respectively. Further details can be found in Table 7.

Finally, in the execution time comparison (Supplementary Materials Figure S4), the RR and RRE methods were the fastest, averaging 0.451 s. The ML method followed with a slightly higher time of 0.55 s. In contrast, the MRG, RRBE, MRGE, and MRG-ML methods were significantly slower, with execution times of 44.08 s, 51.594 s, 51.696 s, and 62.824 s, respectively. See Table 7 for additional details.

### 3.5. Across Data

**Figure 5:**
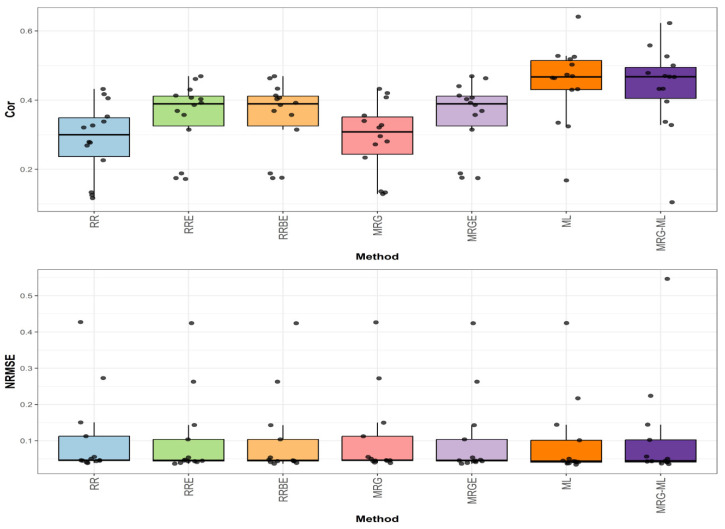
Box plots of Pearson’s correlation (Cor) and normalized root mean square error (NRMSE) across all datasets are presented in the top and bottom graphs, respectively. Each black dot represents a data point per partition trait. A comparison is presented for the 7 evaluated methods.

Figure 5 summarizes the prediction performance across all the datasets used in this study in terms of NRMSE and Cor.

The first comparison (Figure 5) reveals that the ML method achieved the highest average correlation value of 0.471, establishing our performance benchmark. The MRG-ML method followed closely with an average correlation value of 0.453, representing a modest 3.974% decrease. The RRE, RRBE, and MRGE methods demonstrated identical average correlation values of 0.379, showing a more substantial 24.274% reduction compared to ML. The poorest performers were the MRG and RR methods, with average correlation values of 0.306 and 0.301, respectively, lagging behind ML by 53.922% and 56.478%. Detailed values are available in Table 8.

For the second comparison (Figure 5), examining prediction error, the ML method again showed optimal performance with the lowest average NRMSE value of 3.804. The RRBE, MRG, and RR methods all performed comparably and were close, with average NRMSE values of 3.814, 3.815, and 3.816, representing an increase of 0.263%, 0.289%, and 0.315%, respectively. The RRE, MRGE, and MRG-ML methods also exhibited slightly higher errors with average NRMSE values of 3.824, 3.829, and 3.839, corresponding to increases of 0.526%, 0.657%, and 0.920%, respectively. Further details can be found in Table 8.

**Table 8:** Prediction performance across the 14 datasets in terms of normalized root mean square error (NRMSE), Pearson’s correlation (Cor), and average method execution (Time). NRMSE_SD denotes the standard deviation of NRMSE, Cor_SD denotes the standard deviation of Cor, and Time_SD denotes the standard deviation of Time.

Method	Cor	Cor_SD	NRMSE	NRMSE_SD	Time	Time_SD
RR	0.301	0.174	3.816	66.641	1.322	1.230
RRE	0.379	0.165	3.824	66.870	1.322	1.230
RRBE	0.379	0.163	3.814	66.678	160.407	154.344
MRG	0.306	0.172	3.815	66.634	153.638	160.082
MRGE	0.379	0.164	3.829	66.956	186.612	199.197
ML	0.471	0.167	3.804	66.642	1.369	0.800
MRG-ML	0.453	0.180	3.839	67.039	163.233	99.604
RR	0.301	0.174	3.816	66.641	1.322	1.230
RRE	0.379	0.165	3.824	66.870	1.322	1.230

The execution time analysis (Supplementary Materials Figure S5) showed stark contrasts between methods. The RR and RRE methods were by far the fastest, averaging just 1.322 s per dataset. The ML method was marginally slower at 1.369 s, maintaining excellent computational efficiency. In contrast, the remaining methods required substantially more processing time: MRG averaged 153.638 s, followed by RRBE at 160.407 s, MRG-ML at 163.233 s, and MRGE at 186.612 s. This demonstrates a clear trade-off between prediction accuracy and computational speed, with RR/RRE offering the best balance for time-sensitive applications while ML provides optimal accuracy with only minimal additional computational cost.

### 3.6. Summary of Results

To facilitate comparison across all 14 datasets used in this study—including those detailed in the Supplementary Materials. Furthermore, Table 9 presents a consolidated summary of the top two performing methods per dataset based on Pearson’s correlation and execution time. The ML method emerged as the overall best performer, consistently achieving the highest correlation values in most datasets, while the MRG-ML hybrid method often ranked second. Execution times varied significantly, with RR and RRE being the fastest and methods like MRG-ML and MRGE requiring substantially more time. These findings underscore the balance between predictive accuracy and computational cost, offering practical insights for selecting lambda optimization strategies in high-dimensional genomic prediction tasks.

**Table 9:** Summary of results, including highest and second-highest correlation and execution time in seconds for the 14 datasets and methods.

Dataset	Top Performing Method	Second Best Method	Highest Correlation	Second Highest Correlation	Execution Time (Seconds)
Disease	RRBE & MRGE	RRE	0.176	0.172	RR/RRE: 0.739, ML: 0.9; Others: 74–93
EYT_1	ML	MRG-ML	0.474	0.396	RR/RRE: 1.03; ML: 1.366; Others: 112–160
Groundnut	ML	MRG-ML	0.641	0.622	RR/RRE: 0.453; ML: 0.803; Others: 45–86
Japonica	MRG-ML	ML	0.558	0.528	RR/RRE: 0.451; ML: 0.55; Others: 44–63
Average (All)	ML	MRG-ML	0.471	0.453	RR/RRE: 1.322; ML: 1.369; Others: 153–186
EYT_2	ML	MRG-ML	0.462	0.45	RR/RRE: ~1; ML: ~1.3; Others: 110–165
EYT_3	ML	MRG-ML	0.491	0.47	RR/RRE: ~1; ML: ~1.4; Others: 115–160
Indica	ML	MRG-ML	0.503	0.489	RR/RRE: ~1; ML: ~1.35; Others: 120–168
Maize	ML	MRG-ML	0.488	0.476	RR/RRE: ~1.1; ML: ~1.4; Others: 122–170
Wheat_1	ML	MRG-ML	0.47	0.455	RR/RRE: ~1.2; ML: ~1.5; Others: 125–175
Wheat_3	ML	MRG-ML	0.465	0.45	RR/RRE: ~1.1; ML: ~1.45; Others: 120–160
Wheat_4	ML	MRG-ML	0.476	0.462	RR/RRE: ~1.2; ML: ~1.4; Others: 130–165
Wheat_5	ML	MRG-ML	0.48	0.465	RR/RRE: ~1.2; ML: ~1.38; Others: 125–170
Wheat_6	ML	MRG-ML	0.473	0.46	RR/RRE: ~1.2; ML: ~1.36; Others: 126–169

Across all datasets, the ML method demonstrated the highest average correlation (0.471), outperforming all other approaches, including the second-best method, MRG-ML, by 3.82% on average. In several datasets (e.g., Groundnut, EYT_3, Indica), the advantage was even more pronounced, exceeding 10% improvement in predictive correlation over traditional methods like RR and RRE. Moreover, ML achieved this superior accuracy with minimal increase in execution time, making it not only the most accurate but also one of the most computationally efficient options. These results firmly position ML as the preferred method for lambda selection in ridge regression, especially in high-dimensional genomic prediction contexts where both accuracy and speed are critical.

In terms of prediction error as measured by NRMSE, the ML method also consistently achieved the lowest average values across all 14 datasets, confirming its superior predictive calibration in addition to its accuracy. The difference between ML and the other methods was often modest in absolute terms but still meaningful in relative percentage. For example, ML had an average NRMSE of 3.804, while RRBE, MRG, and RR had slightly higher values of 3.814, 3.815, and 3.816, respectively—translating into relative increases of only 0.263% to 0.315%. However, more complex methods such as MRG-ML and MRGE had increases approaching 0.9% over ML. These differences, although subtle, can have important cumulative effects in large-scale genomic selection programs, especially when scaled across multiple traits, environments, or selection cycles. The results reinforce the ML method’s balance between minimizing prediction error and computational efficiency, making it a robust choice for real-world breeding applications.

## 4. Discussion

Ridge regression is a powerful regularization technique particularly well-suited for predictive modeling in high-dimensional settings where the number of predictors (p) exceeds the number of observations (n). In such contexts, traditional linear regression becomes unstable or infeasible due to multicollinearity and overfitting (Hastie, Tibshirani, & Friedman, 2009) [13]. RR addresses these issues by adding a penalty term proportional to the squared magnitude of the coefficients, effectively shrinking them towards zero and reducing variance without eliminating any predictor (Hoerl & Kennard, 1970) [1]. This controlled shrinkage allows the model to retain all predictors while improving generalization to unseen data. In the context of small n and large p, such as in genomic selection or image-based phenotyping, RR is often preferred due to its computational simplicity and robustness (de los Campos et al., 2013) [3]. Its ability to handle correlated predictors and stabilize coefficient estimates makes it particularly effective for continuous response prediction where interpretability may be secondary to predictive accuracy.

One of the central challenges in applying ridge regression lies in the selection of the regularization parameter, lambda (λ), which controls the degree of shrinkage applied to the model coefficients. Choosing an inappropriate value for λ can lead to underfitting when the penalty is too strong or overfitting when it is too weak, ultimately compromising the model’s predictive accuracy (Hastie, Tibshirani, & Friedman, 2009) [13]. Despite the popularity of methods such as cross-validation, generalized cross-validation, and information criteria, there is still no definitive consensus on the optimal strategy for λ selection, particularly in small-sample, high-dimensional settings where these methods can be unstable or computationally intensive (Cawley & Talbot, 2010) [14]. Moreover, in contexts like genomics and other biological sciences, where interpretability and generalization are critical, the importance of accurate hyperparameter tuning becomes even more pronounced (de los Campos et al., 2013) [3]. Thus, the selection of λ remains an open area of research, as new methods continue to emerge aiming to balance bias and variance more effectively under diverse data scenarios.

For this reason, we compared recent methods for selecting the regularization parameter, lambda, in the context of RR. Our results showed that the ML method achieved the highest performance in terms of Pearson’s correlation, followed by the MRG-ML method as the second-best performer. In contrast, the RRE, RRBE, and MRGE methods showed a reduction in approximately 24.27% in Pearson’s correlation compared to ML. The poorest results were observed for the MRG and RR methods, with decreases of 53.92% and 56.48%, respectively, relative to the ML method.

The best-performing method is inspired by the approach used in mixed models, where the regularization parameter lambda is estimated as the ratio of genetic variance to residual variance. However, in the context of RR, accurately estimating these variance components can be challenging. To address this limitation, we proposed evaluating lambda over a grid of values derived from plausible ranges for each variance component. This strategy not only improved prediction performance but also proved to be computationally efficient.

The second-best method is a hybrid approach that combines elements of the MRG and ML methods. A key feature of the MRG component is its design to ensure that the training sets used in cross-validation match the size of the original development dataset. This is achieved by performing the tuning process on a pseudo-development dataset, which is constructed by sampling with replacement from the original data to create a dataset larger than the original. As a result, each cross-validation training set maintains the same size as the development dataset, enhancing the comparability of the training conditions. In practice, the MRG method typically applies less shrinkage than standard tuning procedures and tends to be more conservative, favoring models with milder penalization.

Although our conclusions are not definitive, this study contributes additional empirical evidence on the predictive performance and computational efficiency of existing methods for selecting the regularization parameter in RR. This is particularly important given the ongoing debate and lack of consensus in the literature regarding the most effective strategies for tuning lambda, especially in high-dimensional settings (Hastie, Tibshirani, & Friedman, 2009; Cawley & Talbot, 2010) [13,14]. By systematically comparing both traditional and recently proposed approaches, our findings support a more informed selection of tuning methods, highlighting trade-offs between accuracy and execution time. Such empirical evaluations are essential to guide methodological choices in applied contexts, where the balance between predictive performance and computational cost is critical (de los Campos et al., 2013) [3].

### 4.1. Why Some Methods Perform Better than Others?

Several methodological features explain why certain approaches—especially ML and MRG-ML—outperform others. The ML method uses a biologically motivated grid search based on variance component estimates, allowing it to align shrinkage levels with the underlying signal-to-noise structure in the data. This contrasts with fixed-grid or purely heuristic methods, which may overlook optimal regions in the parameter space. The MRG approach ensures consistent training sample sizes via bootstrap-based pseudo-sampling, reducing variance in λ estimation and improving model stability, especially in small datasets.

Traditional CV methods often bias toward conservative λ values, which can reduce model sensitivity to informative markers. Methods like ML and MRG-ML avoid this pitfall by embedding λ estimation within more flexible and biologically meaningful structures. While some methods achieve good accuracy (e.g., RRBE, MRGE), they incur substantial computational costs. The ML method uniquely balances speed and accuracy, making it highly attractive for large-scale genomic applications. MRG-ML benefits from combining cross-validation robustness with variance-driven flexibility. This synergy can be particularly useful when individual methods alone are insufficient due to data heterogeneity or trait complexity.

From a broader perspective, our findings illustrate important trade-offs among competing methods. The extended grid-based variants (RRE, RRBE, MRGE) benefited from greater flexibility at small λ values but did not match the ML or MRG-ML in terms of predictive performance. Standard RR and MRG remained the most computationally efficient but were consistently the least accurate. These results stress the need to consider both performance metrics and runtime when choosing λ-tuning strategies, especially in high-throughput applications like genomic selection.

This study fills a key gap in the literature by systematically comparing modern λ-tuning strategies using extensive real-world data. Our results provide empirical evidence favoring variance component-based and hybrid tuning approaches over classical cross-validation or grid search alone. We advocate for broader adoption of biologically grounded methods like ML in plant and animal breeding pipelines, as they not only improve prediction accuracy but also respect practical constraints on computational resources.

### 4.2. The Way Forward

Looking ahead, several promising directions emerge from this work. First, future studies should assess the generalizability of these findings across a broader range of traits, especially those that are ordinal, binary, or multi-class, which pose additional modeling challenges. Second, incorporating external sources of information such as pedigree structures, environmental covariates, or omics layers (e.g., transcriptomics or metabolomics) into the tuning process may enhance prediction models. Third, given the increasing availability of computational resources, exploring hybrid and ensemble learning strategies that integrate different tuning philosophies could yield robust solutions across heterogeneous datasets.

Furthermore, investigating the integration of these λ-tuning methods within more complex machine learning architectures—such as deep learning and Bayesian neural networks—would be a valuable extension, particularly in environments with non-linear trait architectures or sparse data. Lastly, efforts should be made to package and distribute these methods in open-source software with user-friendly interfaces to facilitate adoption by practitioners in plant and animal breeding programs.

While the Montesinos-López method demonstrated superior predictive performance and computational efficiency, it is important to recognize certain limitations. For instance, the method’s reliance on variance component estimation assumes that underlying genetic architecture can be captured through additive models, which may not hold in all biological contexts. Furthermore, although the ML and MRG-ML methods performed well across datasets, their utility in more complex data structures involving epistatic effects, dominance, or G×E interactions remains to be explored. Future research should extend this benchmarking to additional genomic models, such as Bayesian neural networks or deep kernel learning, and investigate whether lambda tuning strategies remain effective when integrated into nonlinear architectures. Additionally, testing these methods on traits with non-Gaussian distributions (e.g., ordinal, binary) will broaden their applicability in real-world breeding programs.

## 5. Conclusions

In this study, we compared two recently proposed methods for selecting the lambda hyperparameter in Ridge regression. These methods were evaluated alongside conventional approaches and three additional alternative techniques. Our results showed that the method introduced by Montesinos-López et al. (2024) [8] consistently outperformed the others in terms of both predictive performance and computational efficiency. The second-best performance was observed when combining the two recently proposed methods. Based on our empirical evaluation using 14 real-world datasets, we found strong evidence that the Montesinos-López et al. (2024) [8] method was significantly superior to the competing methods. This advantage was particularly evident when assessed using Pearson’s correlation, with a more modest improvement observed for the normalized mean squared error (NRMSE). Two key strengths of the Montesinos-López et al. (2024) [8] method are its conceptual simplicity and computational efficiency, which make it a practical and accessible tool for hyperparameter tuning in Ridge regression. Given these findings, we encourage further benchmarking studies to validate and promote the use of this method within the machine learning community.

## Figures and Tables

**Table 1:** Description of the datasets.

Dataset Number	Dataset (Crop and Number)	Cultivars (Number)	Markers (Number)	Traits (Number)
Dataset 1	Disease	438	11,617	4
Dataset 2	EYT_1	766	2038	4
Dataset 3	EYT_2	775	2038	4
Dataset 4	EYT_3	964	2038	4
Dataset 5	Groundnut	318	8268	4
Dataset 6	Indica	327	16,383	4
Dataset 7	Japonica	320	16,383	4
Dataset 8	Maize	722	54,113	1
Dataset 9	Wheat_1	1301	5741	1
Dataset 10	Wheat_2	1403	5741	1
Dataset 11	Wheat_3	1275	5741	1
Dataset 12	Wheat_4	1388	5741	1
Dataset 13	Wheat_5	1398	5741	1
Dataset 14	Wheat_6	1277	5741	1

**Table 2:** Nomenclature Table with Equations.

Symbol	Definition	Equation (If Applicable)
y	Vector of observed response values	
ŷ_i_	Predicted value for the i-th observation	
***X***	Design matrix of predictor variables	
**y**	Response vector	
β	Vector of regression coefficients	
ε	Random error term (E[ε] = 0)	
λ	Regularization parameter	
n	Number of observations	
p	Number of predictors	
ȳ	Mean of observed response values	
$\sigma_{e}^{2}$	Variance of the residuals	
$\sigma_{\beta }^{2}$	Variance of regression coefficients	
Cor	Pearson’s correlation	Cor = cov(y, ŷ)/(σ_γ_ σ_ŷ)
NRMSE	Normalized root mean square error	NRMSE = √[Σ(y_i_ − ŷ_i_)^2^/n]/ȳ
Time	Execution time (seconds)	

**X** = matrix; **y** = vector.

**Table 3:** Comparison of Lambda Tuning Strategies in Ridge Regression.

Method	Type of λ Selection	Shrinkage Regularization	Data-Driven Adaptation	Requires Prior or Extra Model	Main Advantage
RR	Fixed grid or CV	Yes	No	No	Simple, fast
RRE	Empirical estimation from data	Yes	Yes	No	Automatically adapts λ
RRBE	Bayesian estimation	Yes	Yes	Yes	Uncertainty modeled in λ
ML	Model likelihood-based tuning	Yes	Yes	No	Improves likelihood fit
MRG	Modified ridge from genetic structure (MRG)	Yes	Yes	Yes (G structure)	Integrates marker/pedigree info
MRG-ML	Hybrid of MRG + ML	Yes	Yes	Yes (G + ML)	Combines MRG robustness with ML tuning
MRGE	MRG + Empirical	Yes	Yes	Yes (G)	Combines MRG with empirical data fit

## Data Availability

The data used in this study is available at https://github.com/osval78/Refaning_Penalized_Regression (accessed on 1 January 2025).

## Supplementary Materials

The following supporting information can be downloaded at: https://www.mdpi.com/article/10.3390/genes16060618/s1, Table S1. Prediction performance in terms of normalized root mean square error (NRMSE), Pearson’s correlation (Cor) and average execution of model (Time) for the EYT_2 dataset. NRMSE_SD denotes the standard deviation of NRMSE, Cor_SD denotes the standard deviation of Cor and Time_SD denotes the standard deviation of Time. Table S2. Prediction performance in terms of normalized root mean square error (NRMSE), Pearson’s correlation (Cor) and average execution of model (Time) for the EYT_3 dataset. NRMSE_SD denotes the standard deviation of NRMSE, Cor_SD denotes the standard deviation of Cor and Time_SD denotes the standard deviation of Time. Table S3. Prediction performance in terms of normalized root mean square error (NRMSE), Pearson’s correlation (Cor) and average execution of model (Time) for the Indica dataset. NRMSE_SD denotes the standard deviation of NRMSE, Cor_SD denotes the standard deviation of Cor and Time_SD denotes the standard deviation of Time. Table S4. Prediction performance in terms of normalized root mean square error (NRMSE), Pearson’s correlation (Cor) and average execution of model (Time) for the Maize dataset. NRMSE_SD denotes the standard deviation of NRMSE, Cor_SD denotes the standard deviation of Cor and Time_SD denotes the standard deviation of Time. Table S5. Prediction performance in terms of normalized root mean square error (NRMSE), Pearson’s correlation (Cor) and average execution of model (Time) for the Wheat_1 dataset. NRMSE_SD denotes the standard deviation of NRMSE, Cor_SD denotes the standard deviation of Cor and Time_SD denotes the standard deviation of Time. Table S6. Prediction performance in terms of normalized root mean square error (NRMSE), Pearson’s correlation (Cor) and average execution of model (Time) for the Wheat_2 dataset. NRMSE_SD denotes the standard deviation of NRMSE, Cor_SD denotes the standard deviation of Cor and Time_SD denotes the standard deviation of Time. Table S7. Prediction performance in terms of normalized root mean square error (NRMSE), Pearson’s correlation (Cor) and average execution of model (Time) for the Wheat_3 dataset. NRMSE_SD denotes the standard deviation of NRMSE, Cor_SD denotes the standard deviation of Cor and Time_SD denotes the standard deviation of Time. Table S8. Prediction performance in terms of normalized root mean square error (NRMSE), Pearson’s correlation (Cor) and average execution of model (Time) for the Wheat_4 dataset. NRMSE_SD denotes the standard deviation of NRMSE, Cor_SD denotes the standard deviation of Cor and Time_SD denotes the standard deviation of Time. Table S9. Prediction performance in terms of normalized root mean square error (NRMSE), Pearson’s correlation (Cor) and average execution of model (Time) for the Wheat_5 dataset. NRMSE_SD denotes the standard deviation of NRMSE, Cor_SD denotes the standard deviation of Cor and Time_SD denotes the standard deviation of Time. Table S10. Prediction performance in terms of normalized root mean square error (NRMSE), Pearson’s correlation (Cor) and average execution of model (Time) for the Wheat_6 dataset. NRMSE_SD denotes the standard deviation of NRMSE, Cor_SD denotes the standard deviation of Cor and Time_SD denotes the standard deviation of Time. Figure S1. Bar graph of the average execution time of methods (Time) in seconds for the “Disease” dataset. The seven methods issued in this study are compared (RR, RRE, RRBE, MRG, MRGE, ML and MRG-ML). Figure S2. Bar graph of the average execution time of methods (Time) in seconds for the “EYT_1” dataset. The seven methods issued in this study are compared (RR, RRE, RRBE, MRG, MRGE, ML and MRG-ML) Figure S3. Bar graph of the average execution time of methods (Time) in seconds for the “Groundnut” dataset. The seven methods issued in this study are compared (RR, RRE, RRBE, MRG, MRGE, ML and MRG-ML). Figure S4. Bar graph of the average execution time of methods (Time) in seconds for the “Japonica” dataset. The seven methods issued in this study are compared (RR, RRE, RRBE, MRG, MRGE, ML and MRG-ML). Figure S5. Bar graph of the average execution time of methods (Time) across the 14 datasets used in this study. The seven methods issued in this study are compared (RR, RRE, RRBE, MRG, MRGE, ML and MRG-ML). Figure S6. Box plots of Pearson’s correlation (Cor) and normalized root mean square error (NRMSE) for the “EYT_2” dataset are presented in the top and bottom graphs respectively. Each black dot represents a data point per partition and trait. Figure S7. Bar graph of the average execution time of method (Time) in seconds for the “EYT_2” dataset. The seven methods issued in this study are compared (RR, RRE, RRBE, MRG, MRGE, ML and MRG-ML). Figure S8. Box plots of Pearson’s correlation (Cor) and normalized root mean square error (NRMSE) for the “EYT_3” dataset are presented in the top and bottom graphs respectively. Each black dot represents a data point per fold and trait. Figure S9. Bar graph of the average execution time of method (Time) in seconds for the “EYT_3” dataset. The seven methods issued in this study are compared (RR, RRE, RRBE, MRG, MRGE, ML and MRG-ML). Figure S10. Box plots of Pearson’s correlation (Cor) and normalized root mean square error (NRMSE) for the “Indica” dataset are presented in the top and bottom graphs respectively. Each black dot represents a data point per fold and trait. Figure S11. Bar graph of the average execution time of method (Time) in seconds for the “Indica” dataset. The seven methods issued in this study are compared (RR, RRE, RRBE, MRG, MRGE, ML and MRG-ML). Figure S12. Box plots of Pearson’s correlation (Cor) and normalized root mean square error (NRMSE) for the “Maize” dataset are presented in the top and bottom graphs respectively. Each black dot represents a data point per fold and trait. Figure S13. Bar graph of the average execution time of method (Time) in seconds for the “Maize” dataset. The seven methods issued in this study are compared (RR, RRE, RRBE, MRG, MRGE, ML and MRG-ML). Figure S14. Box plots of Pearson’s correlation (Cor) and normalized root mean square error (NRMSE) for the “Wheat_1” dataset are presented in the top and bottom graphs respectively. Each black dot represents a data point per fold and trait. Figure S15. Bar graph of the average execution time of method (Time) in seconds for the “Wheat_1” dataset. The seven methods issued in this study are compared (RR, RRE, RRBE, MRG, MRGE, ML and MRG-ML). Figure S16. Box plots of Pearson’s correlation (Cor) and normalized root mean square error (NRMSE) for the “Wheat_2” dataset are presented in the top and bottom graphs respectively. Each black dot represents a data point per fold and trait. Figure S17. Bar graph of the average execution time of method (Time) in seconds for the “Wheat_2” dataset. The seven methods issued in this study are compared (RR, RRE, RRBE, MRG, MRGE, ML and MRG-ML). Figure S18. Box plots of Pearson’s correlation (Cor) and normalized root mean square error (NRMSE) for the “Wheat_3” dataset are presented in the top and bottom graphs respectively. Each black dot represents a data point per fold and trait. Figure S19. Bar graph of the average execution time of method (Time) in seconds for the “Wheat_3” dataset. The seven methods issued in this study are compared (RR, RRE, RRBE, MRG, MRGE, ML and MRG-ML). Figure S20. Box plots of Pearson’s correlation (Cor) and normalized root mean square error (NRMSE) for the “Wheat_4” dataset are presented in the top and bottom graphs respectively. Each black dot represents a data point per fold and trait. Figure S21. Bar graph of the average execution time of method (Time) in seconds for the “Wheat_4” dataset. The seven methods issued in this study are compared (RR, RRE, RRBE, MRG, MRGE, ML and MRG-ML). Figure S22. Box plots of Pearson’s correlation (Cor) and normalized root mean square error (NRMSE) for the “Wheat_5” dataset are presented in the top and bottom graphs respectively. Each black dot represents a data point per fold and trait. Figure S23. Bar graph of the average execution time of method (Time) in seconds for the “Wheat_5” dataset. The seven methods issued in this study are compared (RR, RRE, RRBE, MRG, MRGE, ML and MRG-ML). Figure S24. Box plots of Pearson’s correlation (Cor) and normalized root mean square error (NRMSE) for the “Wheat_6” dataset are presented in the top and bottom graphs respectively. Each black dot represents a data point per fold and trait. Figure S25. Bar graph of the average execution time of method (Time) in seconds for the “Wheat_6” dataset. The seven methods issued in this study are compared (RR, RRE, RRBE, MRG, MRGE, ML and MRG-ML)
