# Supplementary material for: Tuning Matters: Comparing Lambda Optimization Approaches for Ridge Regression in Genomic Prediction
Source: Genes (Basel). 2025 May 23;16(6):618. doi: 10.3390/genes16060618 (PMC12193363; doi:10.3390/genes16060618)
Supplement: Supplementary file 1 [file genes-16-00618-s001.zip › genes-3649335-supplementary.pdf]

Supplementary Material

Tables S1–S10

Table S1. Prediction performance in terms of normalized root mean square error (NRMSE), Pearson's correlation (Cor) and average execution of model (Time) for the EYT_2 dataset. NRMSE_SD denotes the standard deviation of NRMSE, Cor_SD denotes the standard deviation of Cor and Time_SD denotes the standard deviation of Time.

Dataset	Method	Cor	Cor_SD	NRMSE	NRMSE _SD	Time	Time_SD
EYT_2	RR	0.279	0.094	0.044	0.013	1.071	0.065
EYT_2	RRE	0.407	0.087	0.042	0.012	1.071	0.065
EYT_2	RRBE	0.407	0.087	0.042	0.012	126.508	2.929
EYT_2	MRG	0.296	0.088	0.044	0.013	115.809	3.048
EYT_2	MRGE	0.407	0.087	0.042	0.012	137.614	2.738
EYT_2	ML	0.519	0.097	0.039	0.011	1.398	0.133
EYT_2	MRG-ML	0.479	0.112	0.044	0.016	159.137	8.239

Table S2. Prediction performance in terms of normalized root mean square error (NRMSE), Pearson's correlation (Cor) and average execution of model (Time) for the EYT_3 dataset. NRMSE_SD denotes the standard deviation of NRMSE, Cor_SD denotes the standard deviation of Cor and Time_SD denotes the standard deviation of Time.

Dataset	Method	Cor	Cor_SD	NRMSE	NRMSE _SD	Time	Time_SD
EYT_3	RR	0.277	0.103	0.039	0.013	1.595	0.176
EYT_3	RRE	0.386	0.087	0.037	0.012	1.595	0.176
EYT_3	RRBE	0.386	0.087	0.037	0.012	195.013	20.253
EYT_3	MRG	0.281	0.100	0.039	0.013	175.325	13.474
EYT_3	MRGE	0.386	0.087	0.037	0.012	210.794	20.454

EYT_3	ML	0.503	0.063	0.035	0.011	1.986	0.319
EYT_3	MRG-ML	0.500	0.063	0.036	0.014	246.726	40.189

Table S3. Prediction performance in terms of normalized root mean square error (NRMSE), Pearson's correlation (Cor) and average execution of model (Time) for the Indica dataset. NRMSE_SD denotes the standard deviation of NRMSE, Cor_SD denotes the standard deviation of Cor and Time_SD denotes the standard deviation of Time.

Dataset	Method	Cor	Cor_SD	NRMSE	NRMSE_SD	Time	Time_SD
Indica	RR	0.417	0.26	0.151	0.188	0.469	0.048
Indica	RRE	0.461	0.235	0.144	0.183	0.469	0.048
Indica	RRBE	0.463	0.233	0.143	0.182	54.51	1.076
Indica	MRG	0.421	0.259	0.15	0.187	47.097	0.651
Indica	MRGE	0.463	0.233	0.143	0.182	54.745	1.545
Indica	ML	0.464	0.225	0.144	0.183	0.806	0.146
Indica	MRG-ML	0.468	0.221	0.144	0.181	112.105	34.147

Table S4. Prediction performance in terms of normalized root mean square error (NRMSE), Pearson's correlation (Cor) and average execution of model (Time) for the Maize dataset. NRMSE_SD denotes the standard deviation of NRMSE, Cor_SD denotes the standard deviation of Cor and Time_SD denotes the standard deviation of Time.

Dataset	Method	Cor	Cor_SD	NRMSE	NRMSE_SD	Time	Time_SD
Maize	RR	0.432	0.069	125.493	387.721	0.081	0.013
Maize	RRE	0.431	0.071	125.920	389.055	0.081	0.013
Maize	RRBE	0.433	0.069	125.582	387.93	8.920	0.674
Maize	MRG	0.433	0.069	125.483	387.677	8.117	0.823
Maize	MRGE	0.441	0.069	126.100	389.55	10.353	4.803
Maize	ML	0.430	0.070	125.490	387.724	0.104	0.057
Maize	MRG-ML	0.433	0.070	126.211	390.050	8.246	0.536

Table S5. Prediction performance in terms of normalized root mean square error (NRMSE),

Pearson's correlation (Cor) and average execution of model (Time) for the Wheat_1 dataset. NRMSE_SD denotes the standard deviation of NRMSE, Cor_SD denotes the standard deviation of Cor and Time_SD denotes the standard deviation of Time.

Dataset	Method	Cor	Cor_SD	NRMSE	NRMSE_SD	Time	Time_SD
Wheat_1	RR	0.226	0.090	0.056	0.004	3.197	0.227
Wheat_1	RRE	0.315	0.062	0.054	0.004	3.197	0.227
Wheat_1	RRBE	0.315	0.062	0.054	0.004	404.847	23.121
Wheat_1	MRG	0.234	0.09	0.055	0.004	430.181	44.314
Wheat_1	MRGE	0.315	0.062	0.054	0.004	541.686	40.363
Wheat_1	ML	0.465	0.053	0.050	0.004	2.411	0.114
Wheat_1	MRG-ML	0.467	0.053	0.050	0.004	310.185	26.87

Table S6. Prediction performance in terms of normalized root mean square error (NRMSE), Pearson's correlation (Cor) and average execution of model (Time) for the Wheat_2 dataset. NRMSE_SD denotes the standard deviation of NRMSE, Cor_SD denotes the standard deviation of Cor and Time_SD denotes the standard deviation of Time.

Dataset	Method	Cor	Cor_SD	NRMSE	NRMSE_SD	Time	Time_SD
Wheat_2	RR	0.117	0.151	0.045	0.003	4.384	0.832
Wheat_2	RRE	0.175	0.126	0.045	0.004	4.384	0.832
Wheat_2	RRBE	0.175	0.126	0.045	0.004	530.736	91.183
Wheat_2	MRG	0.129	0.156	0.045	0.003	549.906	79.123
Wheat_2	MRGE	0.175	0.126	0.045	0.004	668.984	106.229
Wheat_2	ML	0.324	0.076	0.043	0.003	2.897	0.486
Wheat_2	MRG-ML	0.328	0.076	0.043	0.003	345.484	55.057

Table S7. Prediction performance in terms of normalized root mean square error (NRMSE), Pearson's correlation (Cor) and average execution of model (Time) for the Wheat_3 dataset.

NRMSE_SD denotes the standard deviation of NRMSE, Cor_SD denotes the standard deviation of Cor and Time_SD denotes the standard deviation of Time.

Dataset	Method	Cor	Cor_SD	NRMSE	NRMSE _SD	Time	Time_SD
Wheat_3	RR	0.327	0.066	0.045	0.002	2.960	0.16
Wheat_3	RRE	0.369	0.052	0.044	0.001	2.960	0.16
Wheat_3	RRBE	0.369	0.052	0.044	0.001	381.584	9.829
Wheat_3	MRG	0.328	0.066	0.045	0.002	375.930	17.632
Wheat_3	MRGE	0.369	0.052	0.044	0.001	465.320	11.555
Wheat_3	ML	0.47	0.059	0.042	0.002	2.393	0.175
Wheat_3	MRG-ML	0.47	0.059	0.042	0.002	284.170	7.389

Table S8. Prediction performance in terms of normalized root mean square error (NRMSE), Pearson’s correlation (Cor) and average execution of model (Time) for the Wheat_4 dataset. NRMSE_SD denotes the standard deviation of NRMSE, Cor_SD denotes the standard deviation of Cor and Time_SD denotes the standard deviation of Time.

Dataset	Method	Cor	Cor_SD	NRMSE	NRMSE _SD	Time	Time_SD
Wheat_4	RR	0.133	0.113	0.046	0.002	4.220	0.582
Wheat_4	RRE	0.188	0.117	0.046	0.002	4.220	0.582
Wheat_4	RRBE	0.188	0.117	0.046	0.002	524.865	90.81
Wheat_4	MRG	0.133	0.113	0.046	0.002	535.818	84.32
Wheat_4	MRGE	0.188	0.117	0.046	0.002	646.604	105.841
Wheat_4	ML	0.335	0.04	0.044	0.002	3.062	0.373
Wheat_4	MRG-ML	0.337	0.038	0.044	0.002	359.147	63.388

Table S9. Prediction performance in terms of normalized root mean square error (NRMSE), Pearson’s correlation (Cor) and average execution of model (Time) for the Wheat_5 dataset.

NRMSE_SD denotes the standard deviation of NRMSE, Cor_SD denotes the standard deviation of Cor and Time_SD denotes the standard deviation of Time.

Dataset	Method	Cor	Cor_SD	NRMSE	NRMSE _SD	Time	Time_SD
Wheat_5	RR	0.321	0.051	0.040	0.003	4.477	0.758
Wheat_5	RRE	0.357	0.062	0.039	0.003	4.477	0.758
Wheat_5	RRBE	0.357	0.062	0.039	0.003	559.471	100.746
Wheat_5	MRG	0.321	0.051	0.040	0.003	569.491	100.477
Wheat_5	MRGE	0.357	0.062	0.039	0.003	717.663	131.259
Wheat_5	ML	0.432	0.049	0.038	0.003	3.120	0.557
Wheat_5	MRG-ML	0.432	0.049	0.038	0.003	387.625	69.942

Table S10. Prediction performance in terms of normalized root mean square error (NRMSE), Pearson's correlation (Cor) and average execution of model (Time) for the Wheat_6 dataset. NRMSE_SD denotes the standard deviation of NRMSE, Cor_SD denotes the standard deviation of Cor and Time_SD denotes the standard deviation of Time.

Dataset	Method	Cor	Cor_SD	NRMSE	NRMSE_ SD	Time	Time_SD
Wheat_6	RR	0.353	0.048	0.046	0.003	3.128	0.172
Wheat_6	RRE	0.403	0.048	0.044	0.003	3.128	0.172
Wheat_6	RRBE	0.403	0.048	0.044	0.003	381.267	11.786
Wheat_6	MRG	0.355	0.047	0.046	0.003	372.417	13.036
Wheat_6	MRGE	0.403	0.048	0.044	0.003	466.049	13.687
Wheat_6	ML	0.525	0.042	0.041	0.003	2.229	0.143
Wheat_6	MRG-ML	0.527	0.043	0.041	0.003	265.487	6.983

Figures S1–S25

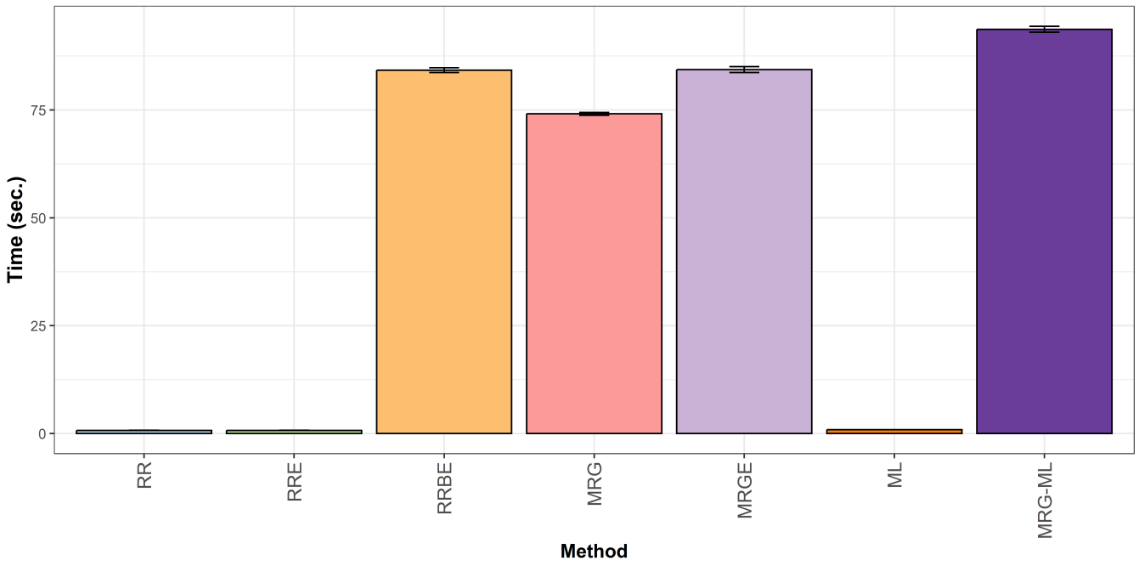

Figure S1. Bar graph of the average execution time of methods (Time) in seconds for the "Disease" dataset. The seven methods issued in this study are compared (RR, RRE, RRBE, MRG, MRGE, ML and MRG-ML).

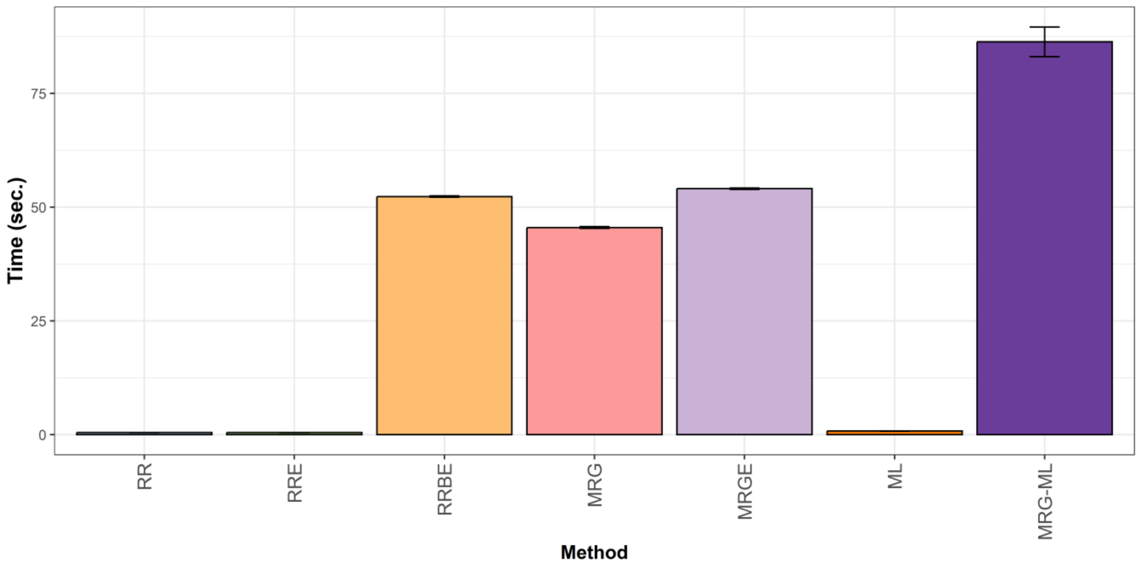

Figure S2. Bar graph of the average execution time of methods (Time) in seconds for the "EYT_1" dataset. The seven methods issued in this study are compared (RR, RRE, RRBE, MRG, MRGE, ML and MRG-ML)

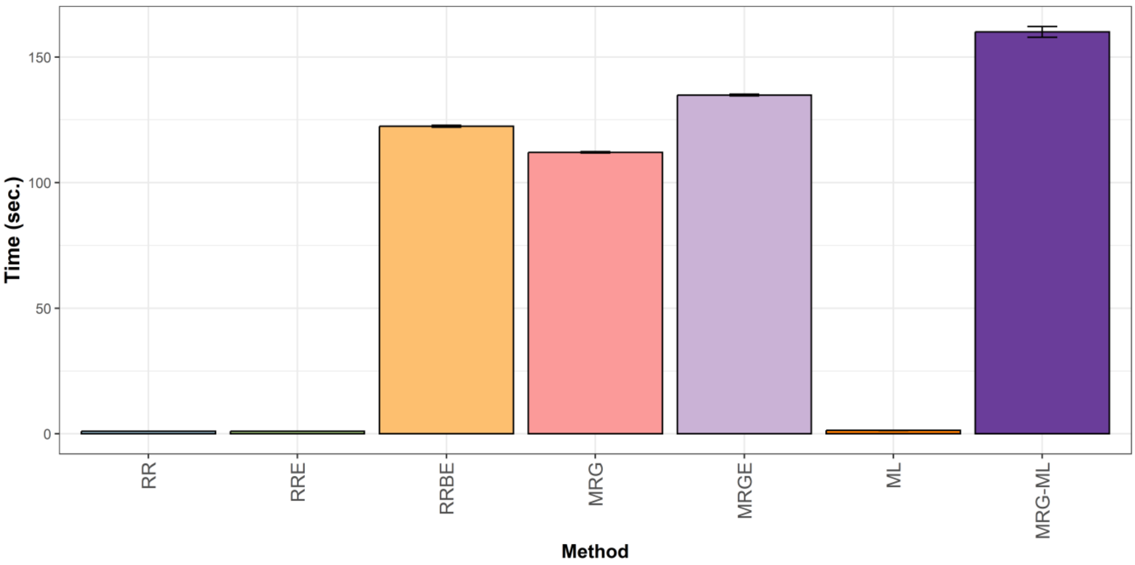

Figure S3. Bar graph of the average execution time of methods (Time) in seconds for the "Groundnut" dataset. The seven methods issued in this study are compared (RR, RRE, RRBE, MRG, MRGE, ML and MRG-ML).

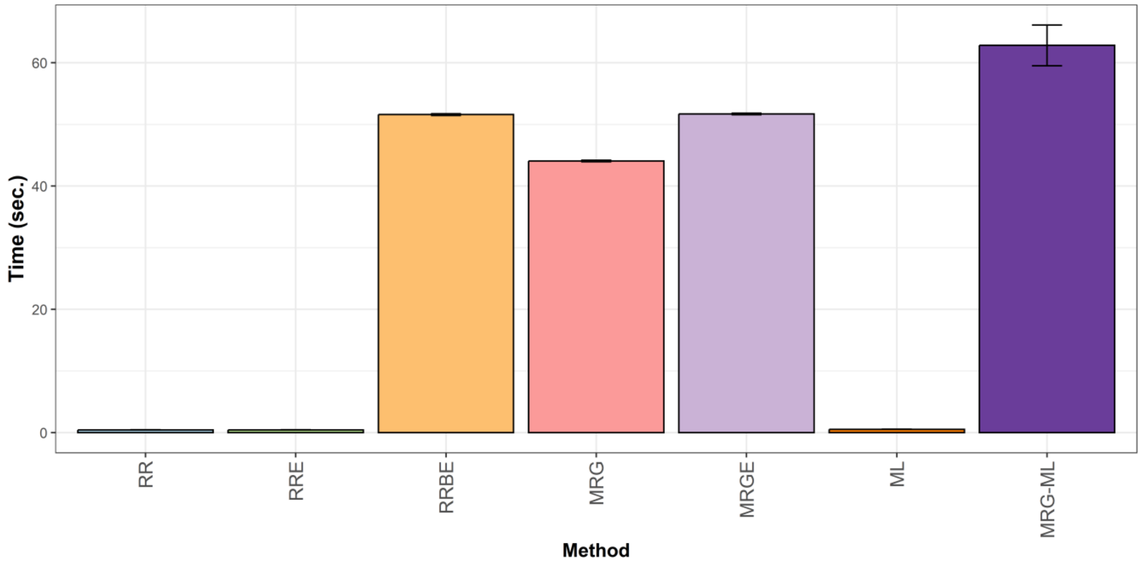

Figure S4. Bar graph of the average execution time of methods (Time) in seconds for the “Japonica” dataset. The seven methods issued in this study are compared (RR, RRE, RRBE, MRG, MRGE, ML and MRG-ML).

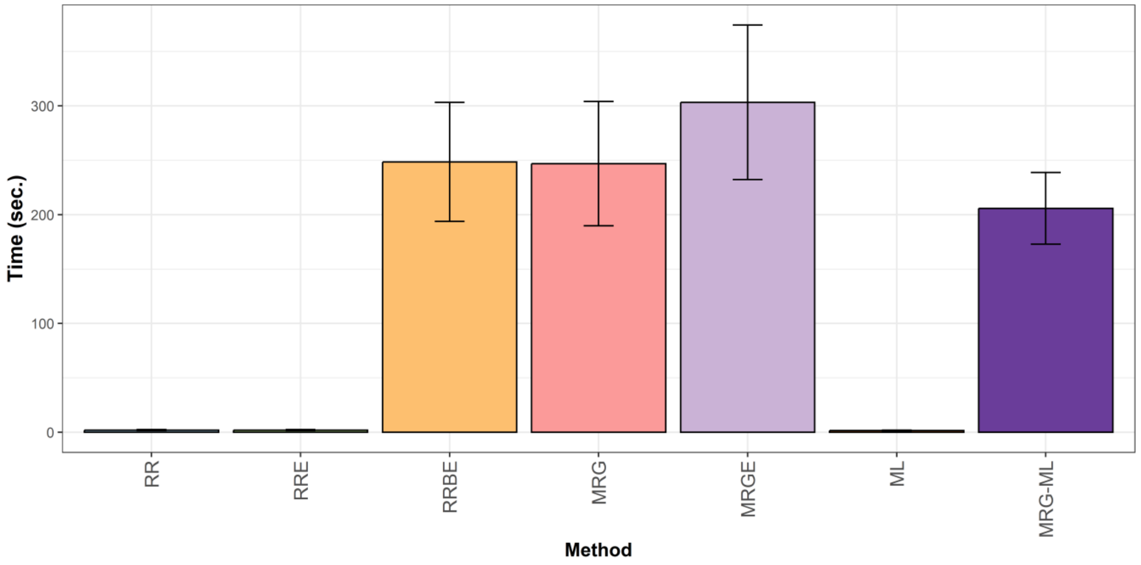

Figure S5. Bar graph of the average execution time of methods (Time) across the 14 datasets used in this study. The seven methods issued in this study are compared (RR, RRE, RRBE, MRG, MRGE, ML and MRG-ML).

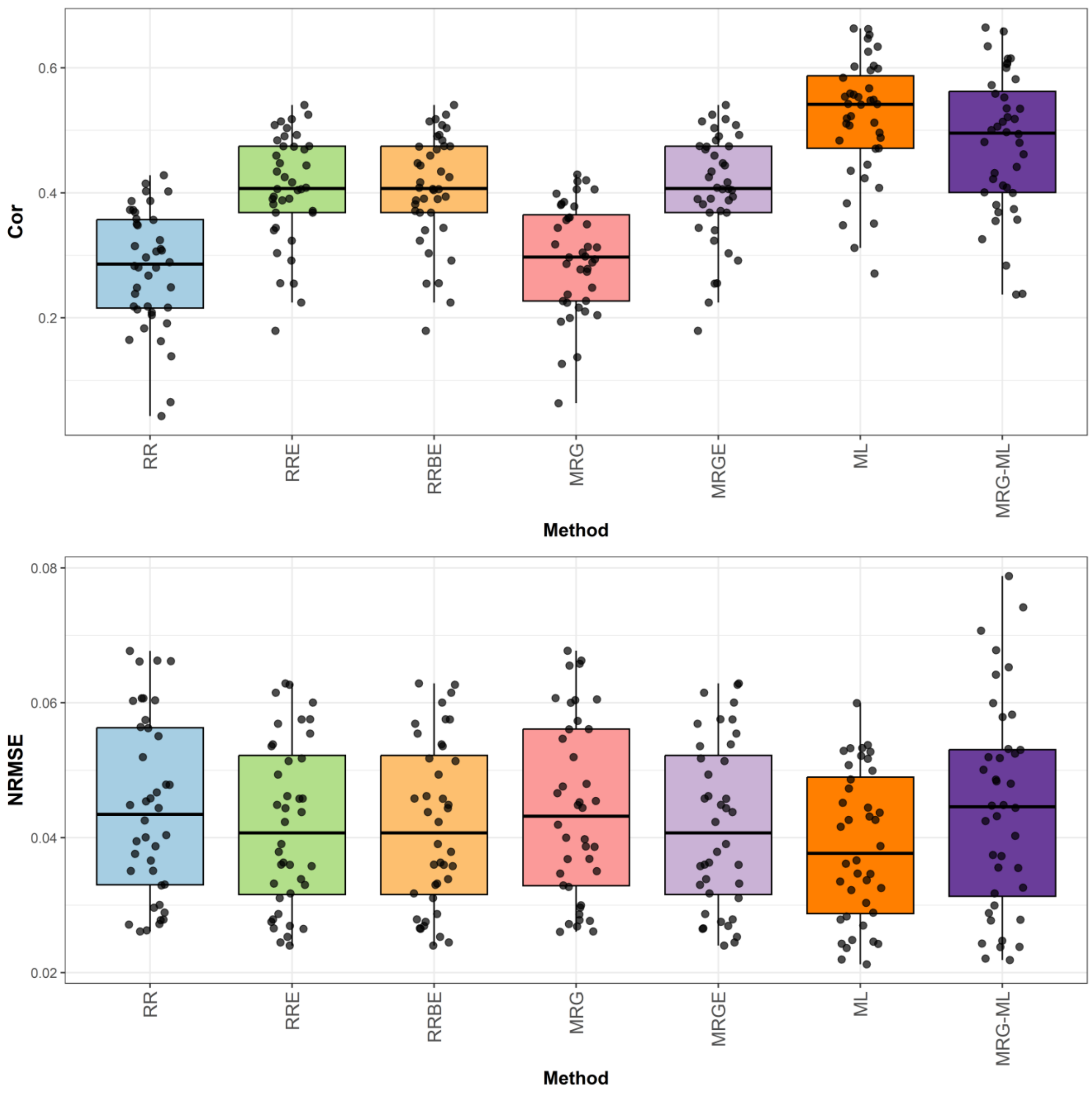

Figure S6. Box plots of Pearson's correlation (Cor) and normalized root mean square error (NRMSE) for the "EYT_2" dataset are presented in the top and bottom graphs respectively. Each black dot represents a data point per partition and trait.

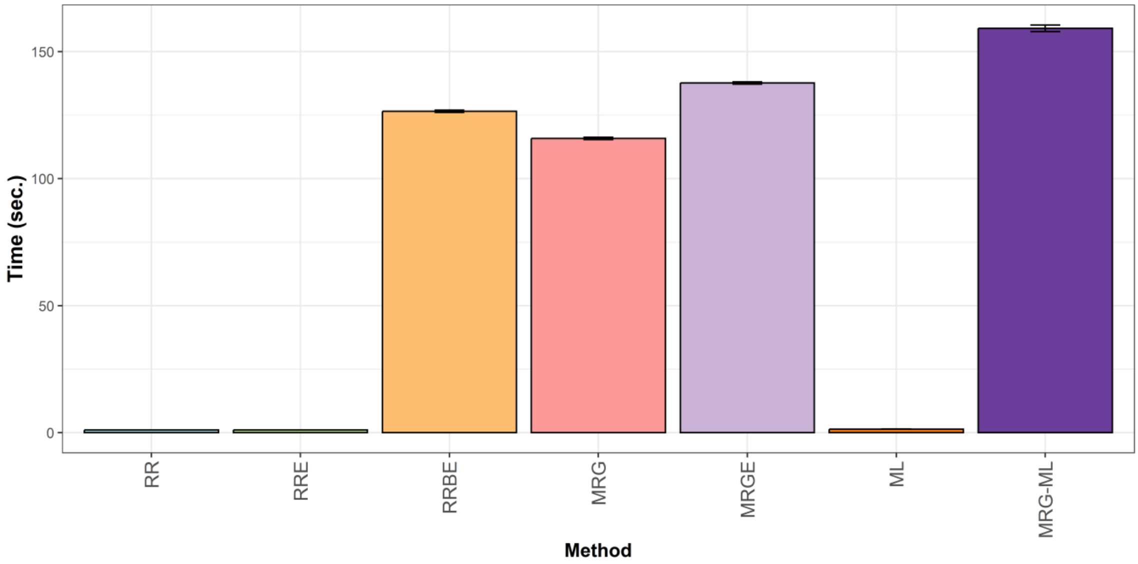

Figure S7. Bar graph of the average execution time of method (Time) in seconds for the "EYT_2" dataset. The seven methods issued in this study are compared (RR, RRE, RRBE, MRG, MRGE, ML and MRG-ML).

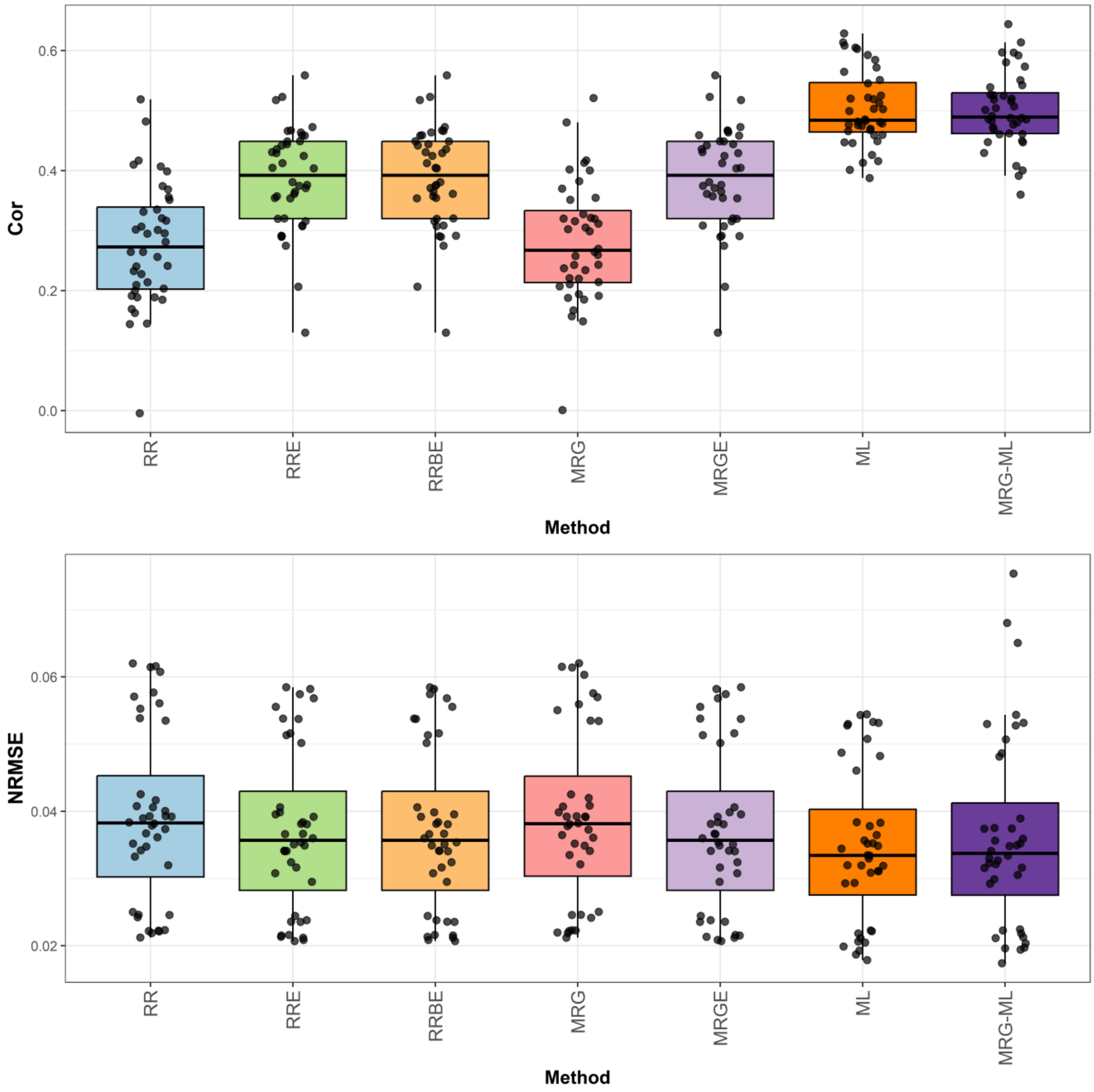

Figure S8. Box plots of Pearson's correlation (Cor) and normalized root mean square error (NRMSE) for the "EYT_3" dataset are presented in the top and bottom graphs respectively. Each black dot represents a data point per fold and trait.

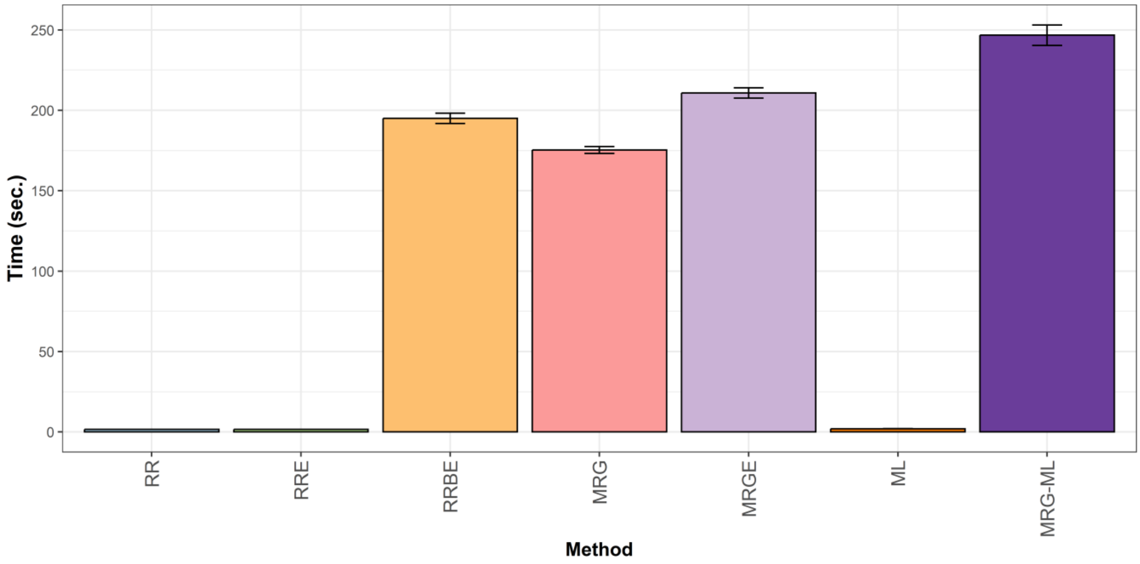

Figure S9. Bar graph of the average execution time of method (Time) in seconds for the "EYT_3" dataset. The seven methods issued in this study are compared (RR, RRE, RRBE, MRG, MRGE, ML and MRG-ML)

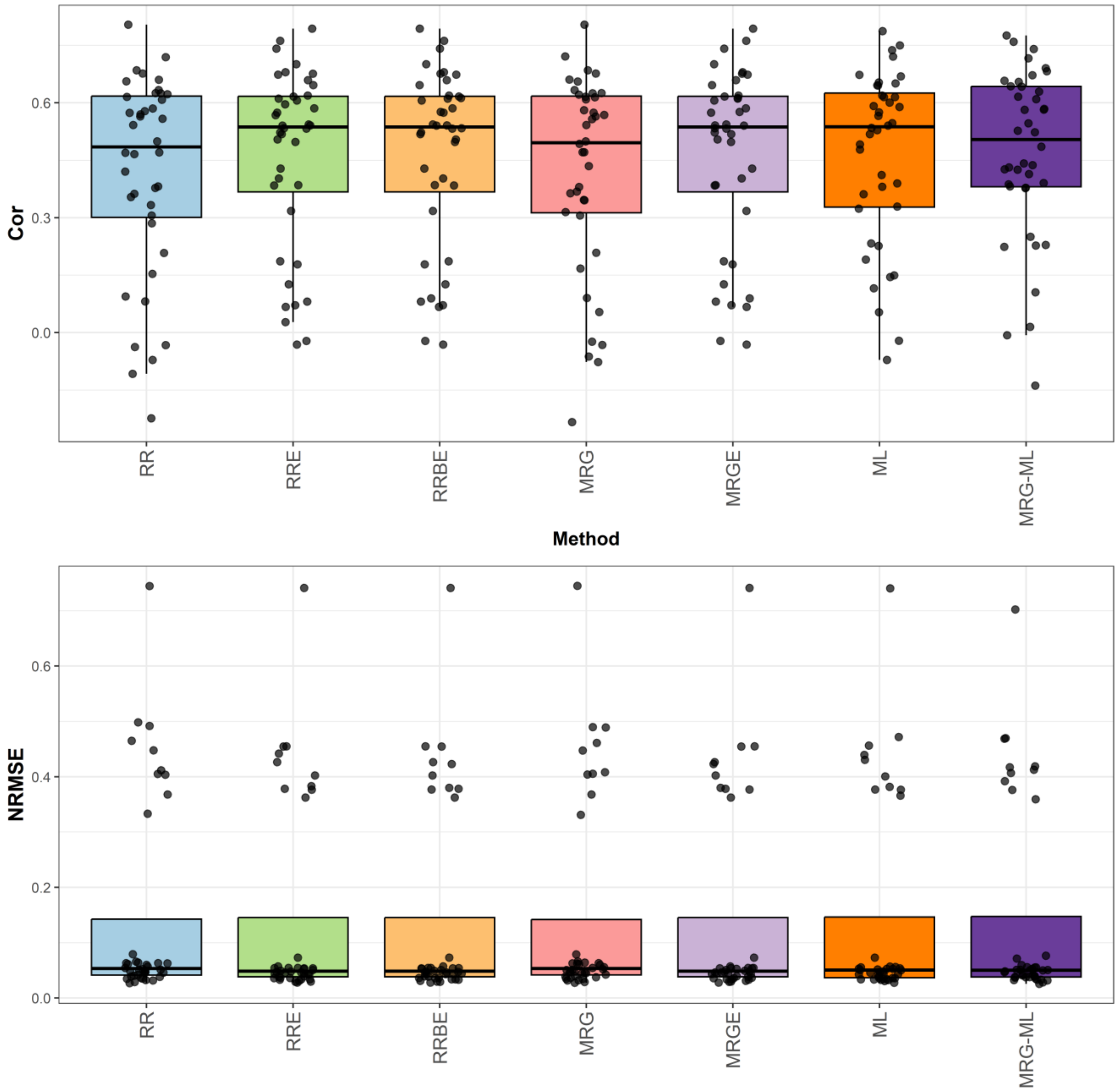

Figure S10. Box plots of Pearson's correlation (Cor) and normalized root mean square error (NRMSE) for the "Indica" dataset are presented in the top and bottom graphs respectively. Each black dot represents a data point per fold and trait.

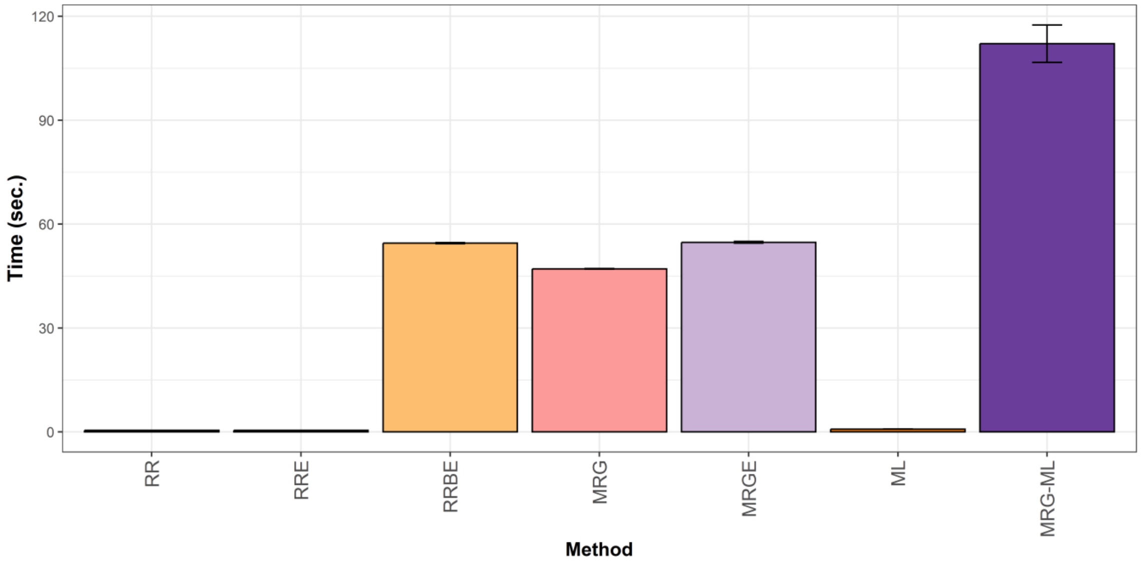

Figure S11. Bar graph of the average execution time of method (Time) in seconds for the "Indica" dataset. The seven methods issued in this study are compared (RR, RRE, RRBE, MRG, MRGE, ML and MRG-ML).

.

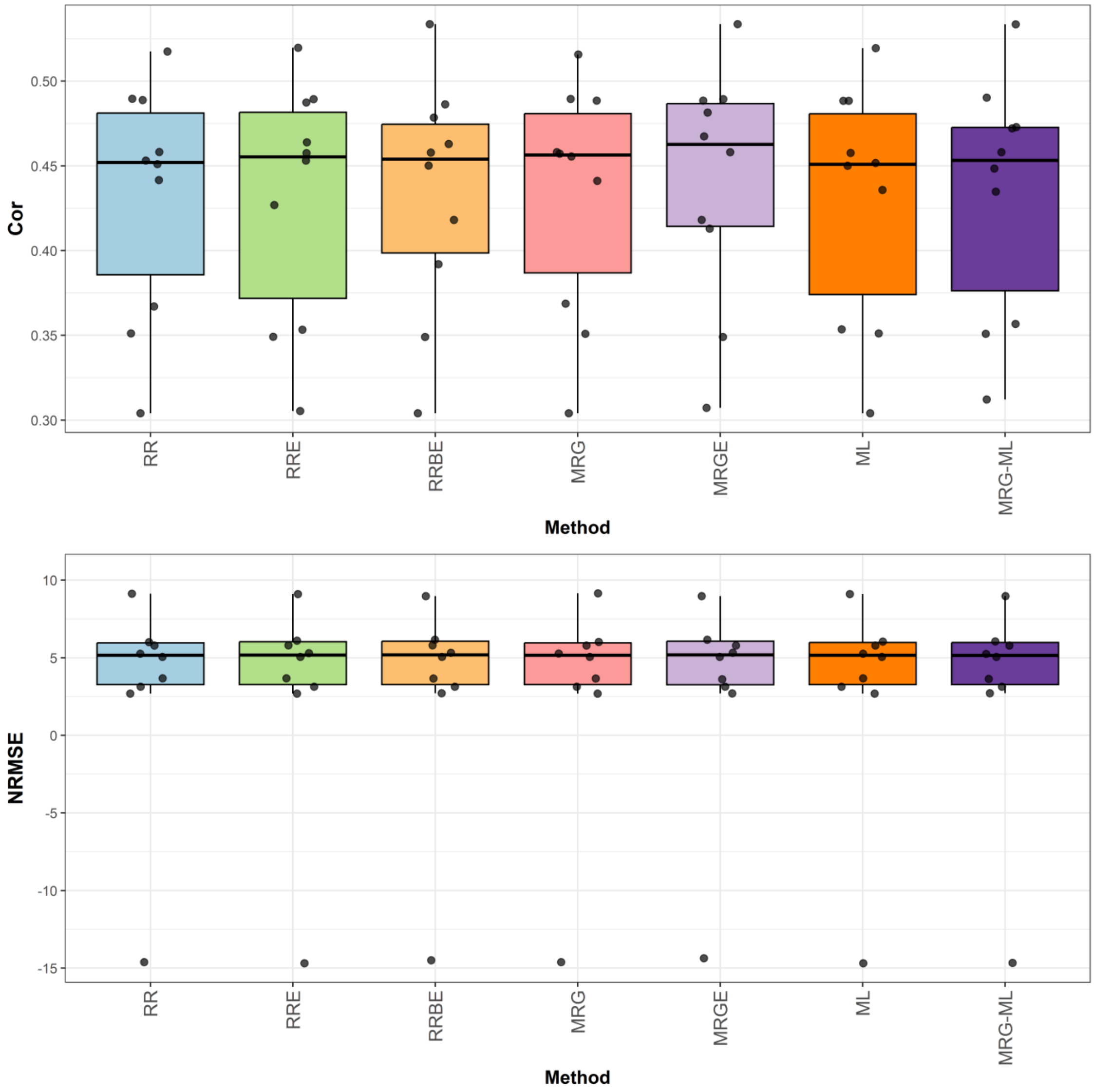

Figure S12. Box plots of Pearson’s correlation (Cor) and normalized root mean square error (NRMSE) for the “Maize” dataset are presented in the top and bottom graphs respectively. Each black dot represents a data point per fold and trait

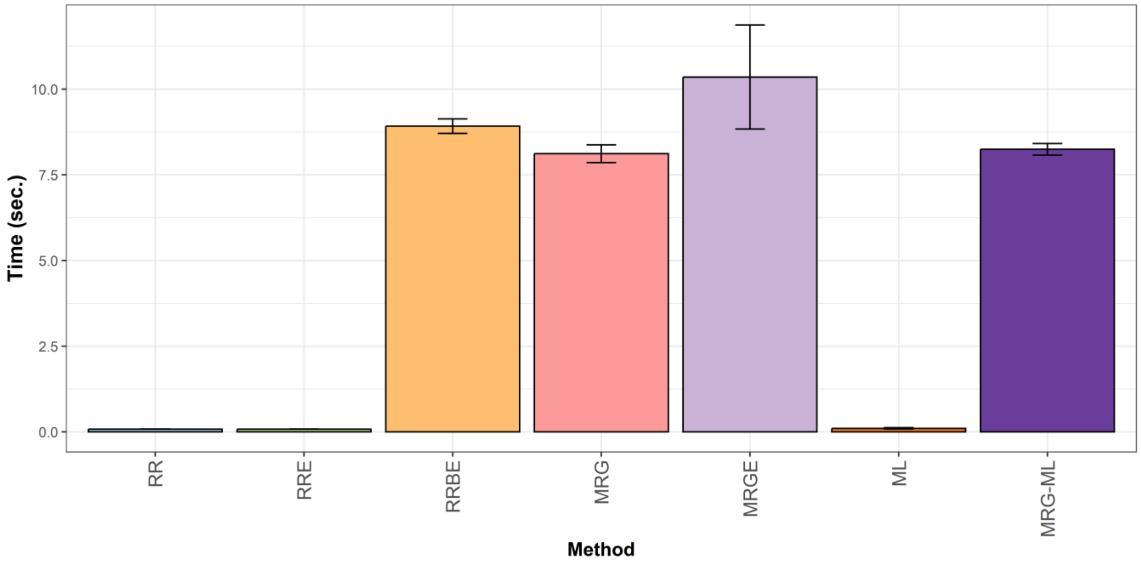

Figure S13. Bar graph of the average execution time of method (Time) in seconds for the "Maize" dataset. The seven methods issued in this study are compared (RR, RRE, RRBE, MRG, MRGE, ML and MRG-ML).

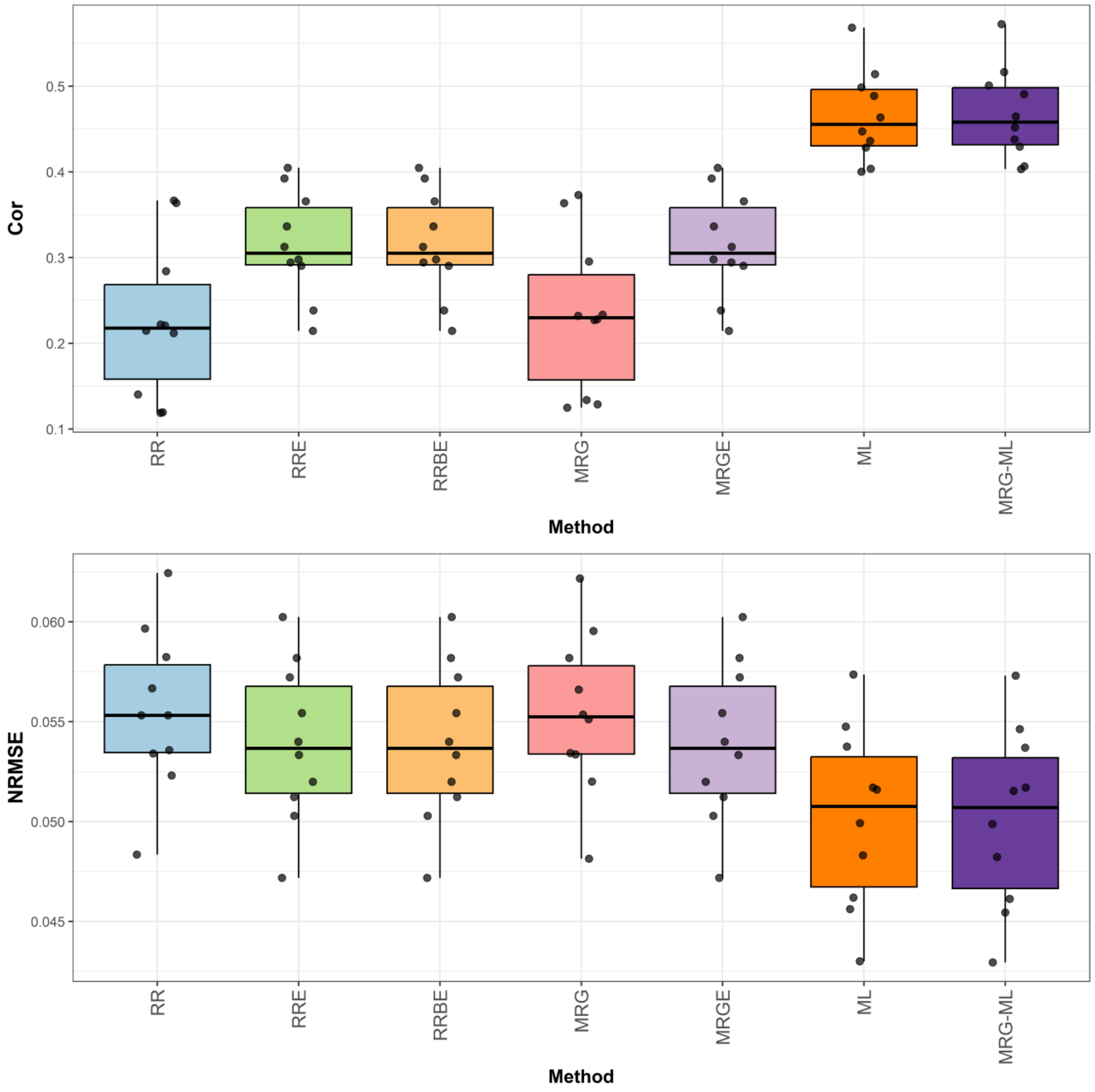

Figure S14. Box plots of Pearson's correlation (Cor) and normalized root mean square error (NRMSE) for the "Wheat_1" dataset are presented in the top and bottom graphs respectively. Each black dot represents a data point per fold and trait.

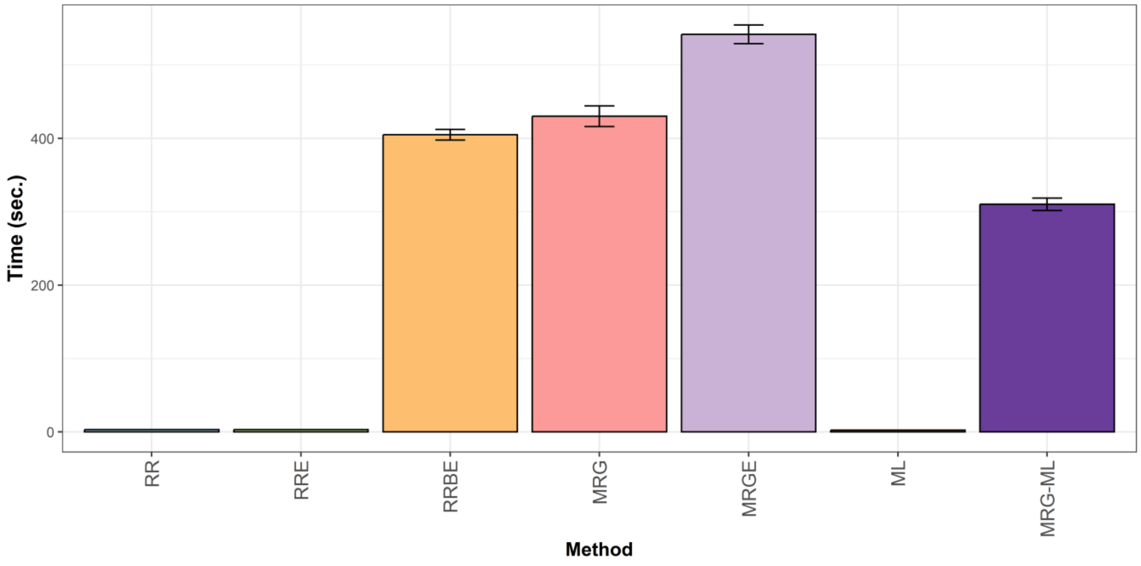

Figure S15. Bar graph of the average execution time of method (Time) in seconds for the "Wheat_1" dataset. The seven methods issued in this study are compared (RR, RRE, RRBE, MRG, MRGE, ML and MRG-ML).

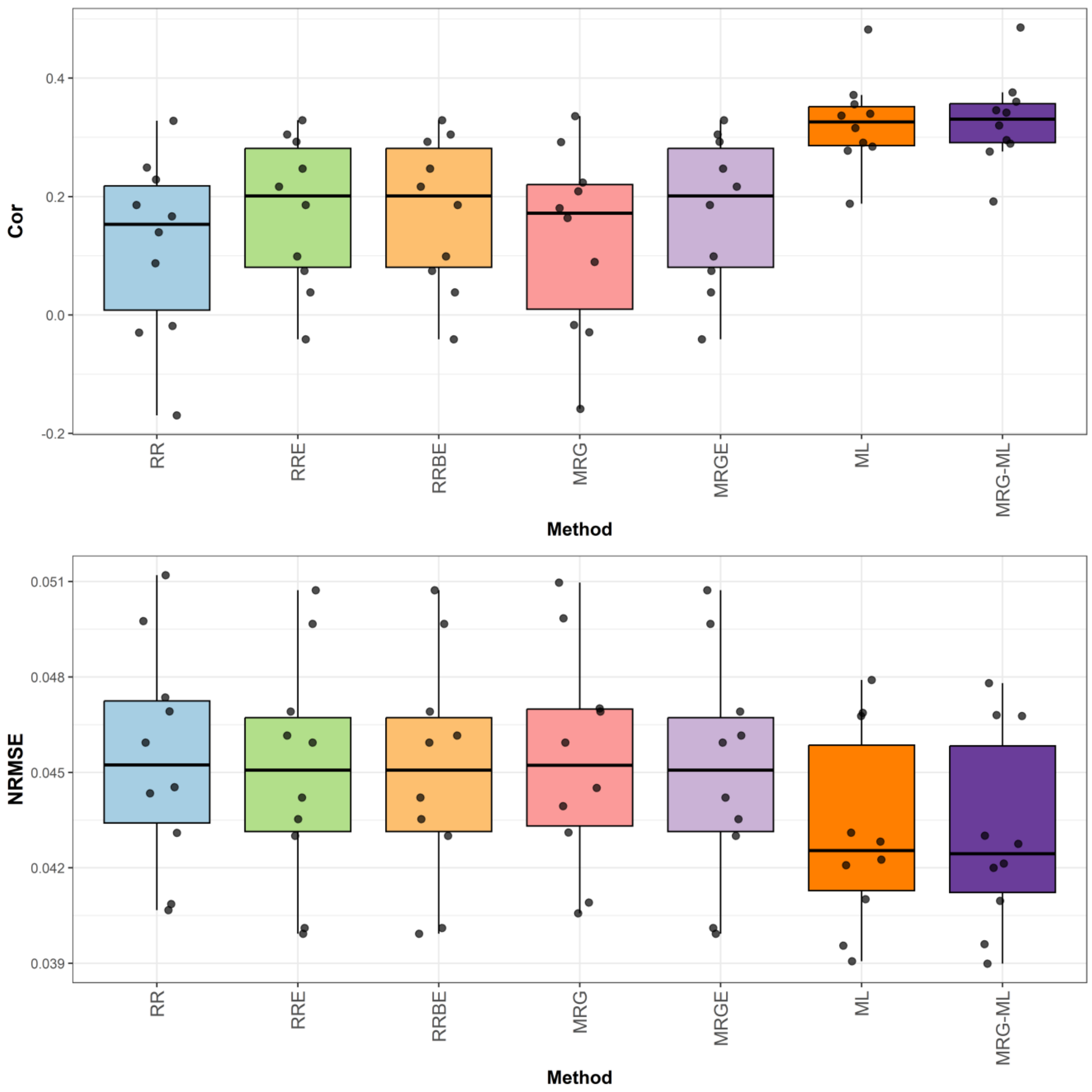

Figure S16. Box plots of Pearson's correlation (Cor) and normalized root mean square error (NRMSE) for the "Wheat_2" dataset are presented in the top and bottom graphs respectively. Each black dot represents a data point per fold and trait.

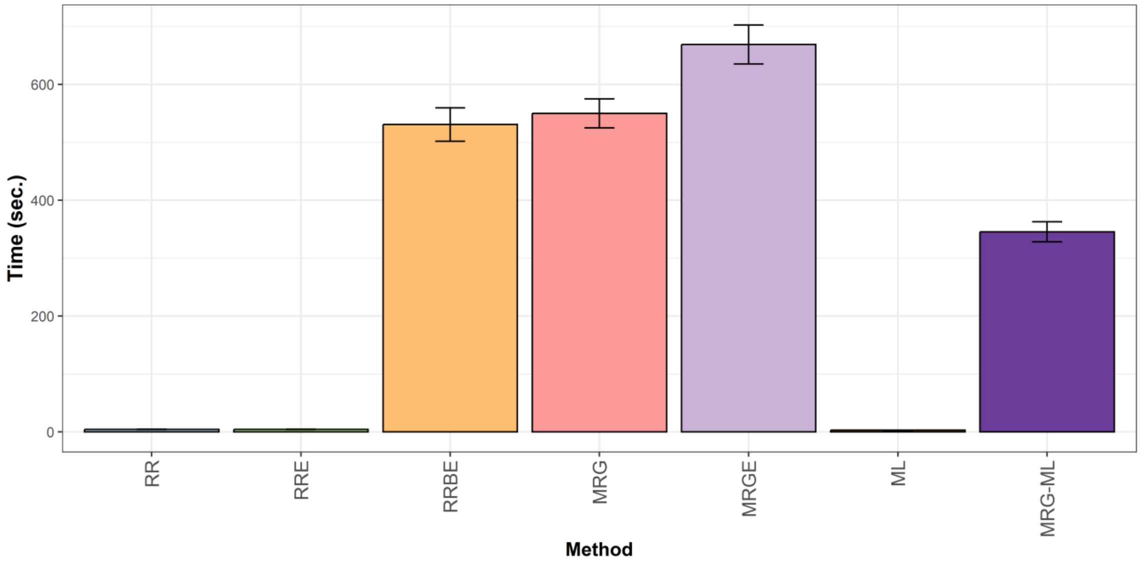

Figure S17. Bar graph of the average execution time of method (Time) in seconds for the "Wheat_2" dataset. The seven methods issued in this study are compared (RR, RRE, RRBE, MRG, MRGE, ML and MRG-ML).

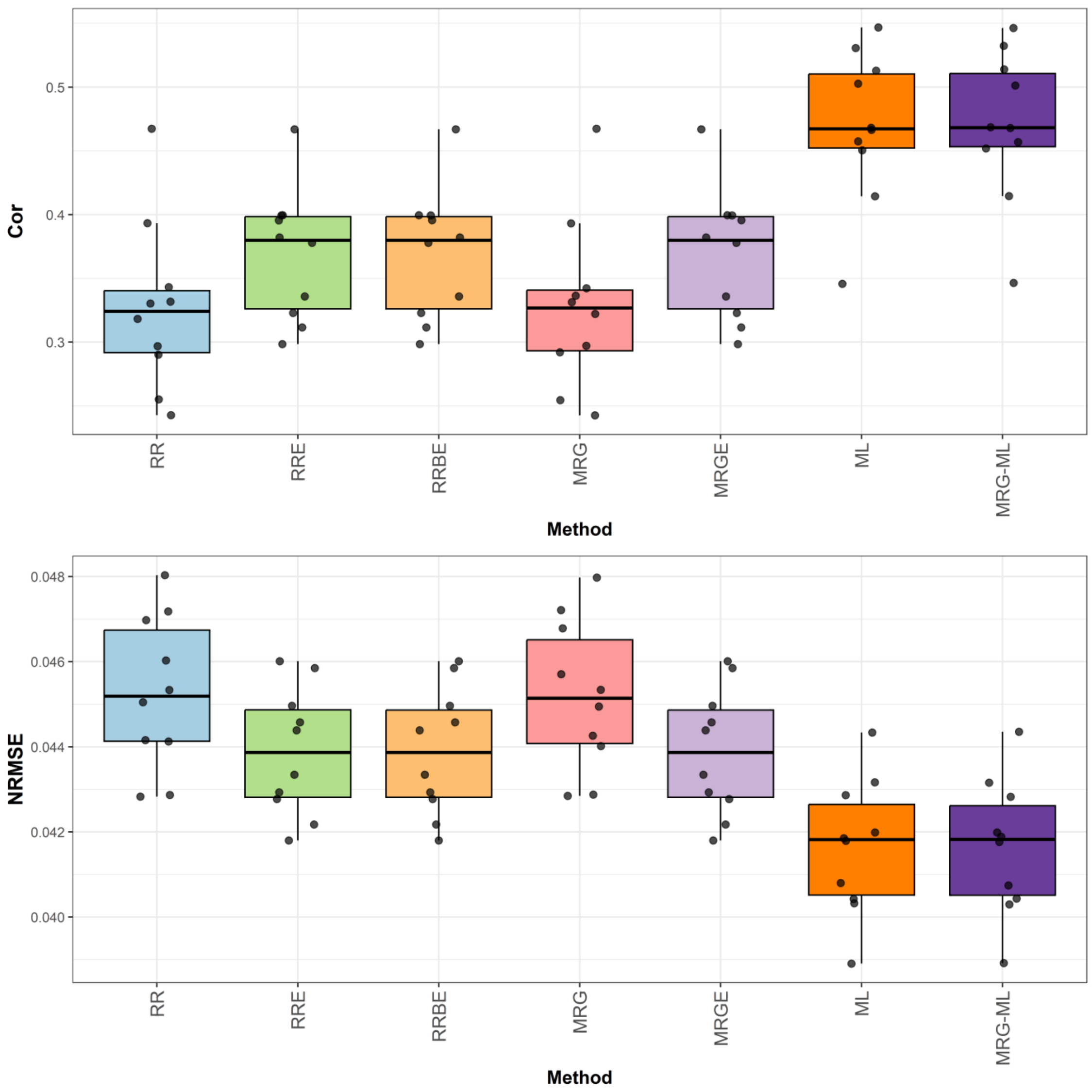

Figure S18. Box plots of Pearson's correlation (Cor) and normalized root mean square error (NRMSE) for the "Wheat_3" dataset are presented in the top and bottom graphs respectively. Each black dot represents a data point per fold and trait.

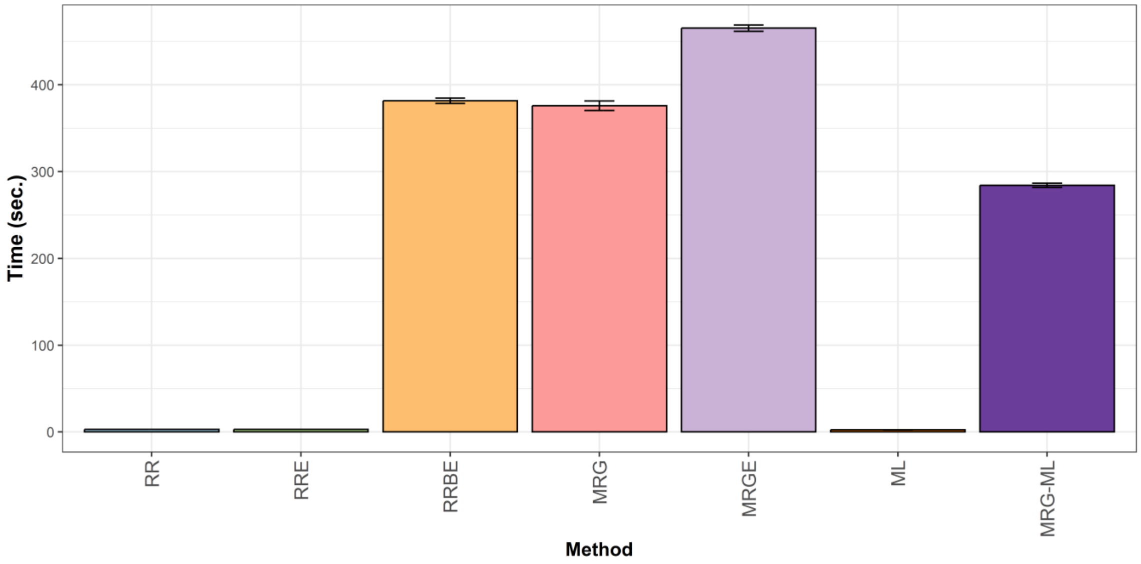

Figure S19. Bar graph of the average execution time of method (Time) in seconds for the "Wheat_3" dataset. The seven methods issued in this study are compared (RR, RRE, RRBE, MRG, MRGE, ML and MRG-ML).

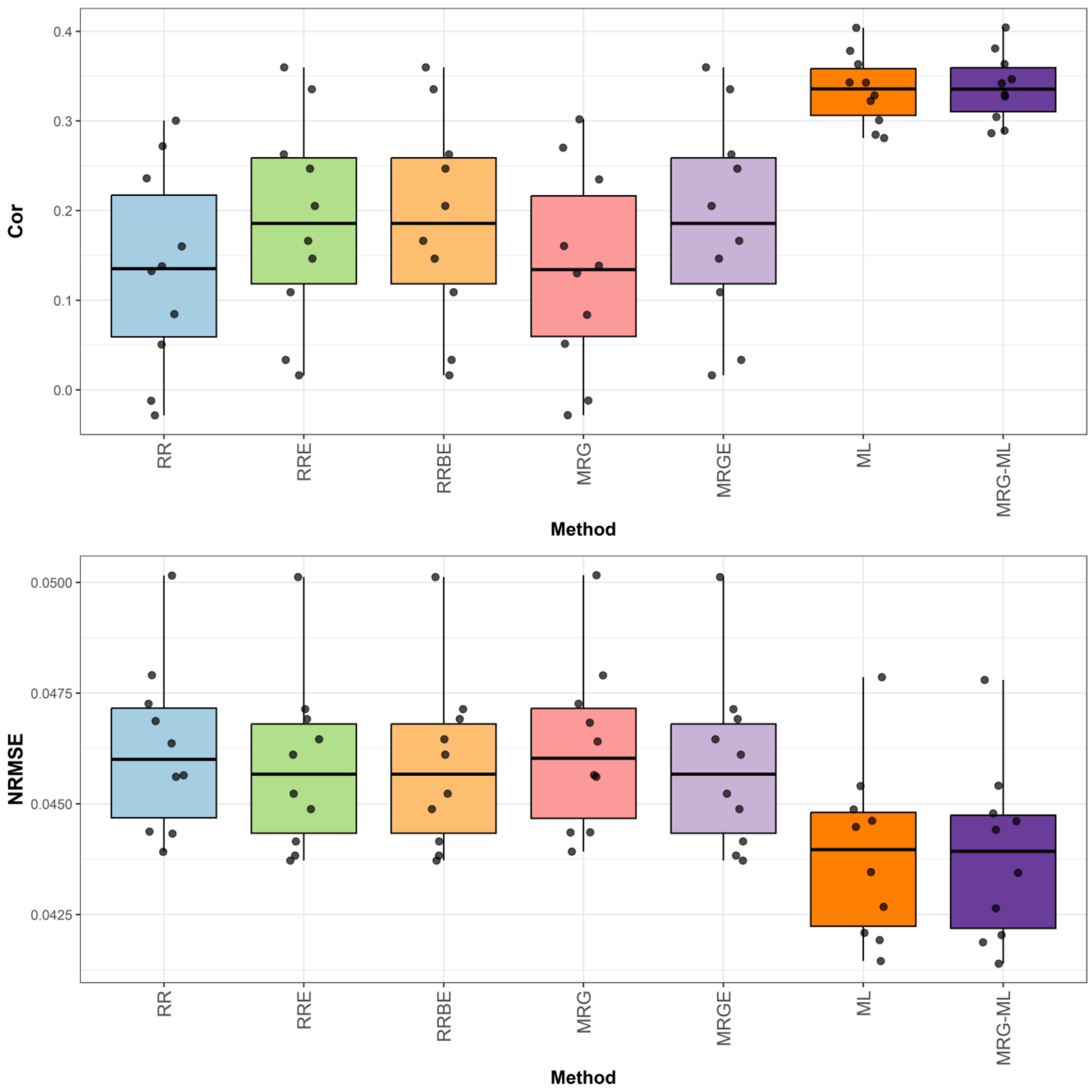

Figure S20. Box plots of Pearson's correlation (Cor) and normalized root mean square error (NRMSE) for the "Wheat_4" dataset are presented in the top and bottom graphs respectively. Each black dot represents a data point per fold and trait.

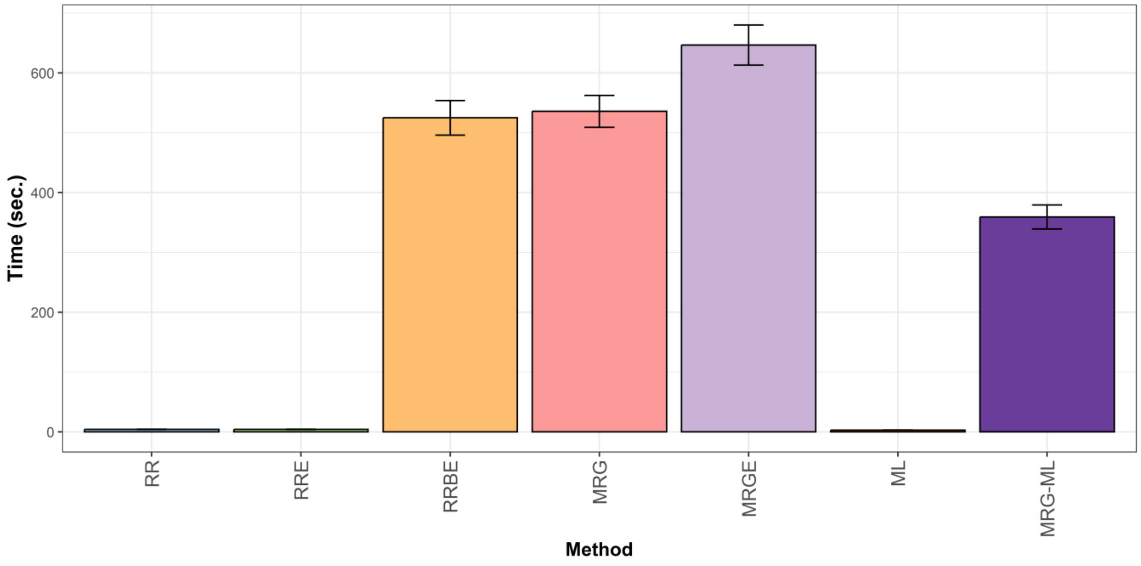

Figure S21. Bar graph of the average execution time of method (Time) in seconds for the “Wheat_4” dataset. The seven methods issued in this study are compared (RR, RRE, RRBE, MRG, MRGE, ML and MRG-ML).

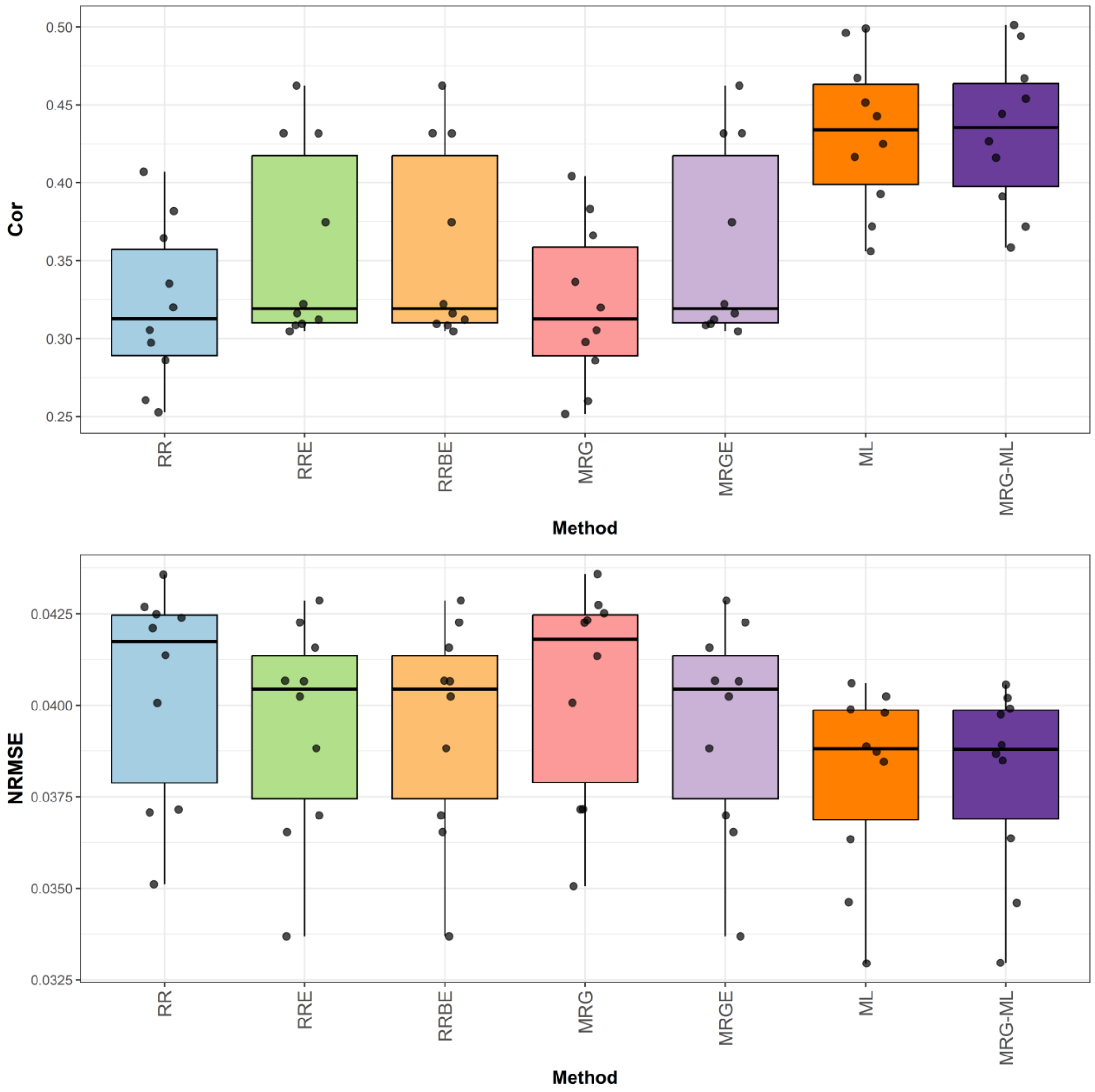

Figure S22. Box plots of Pearson's correlation (Cor) and normalized root mean square error (NRMSE) for the "Wheat_5" dataset are presented in the top and bottom graphs respectively. Each black dot represents a data point per fold and trait.

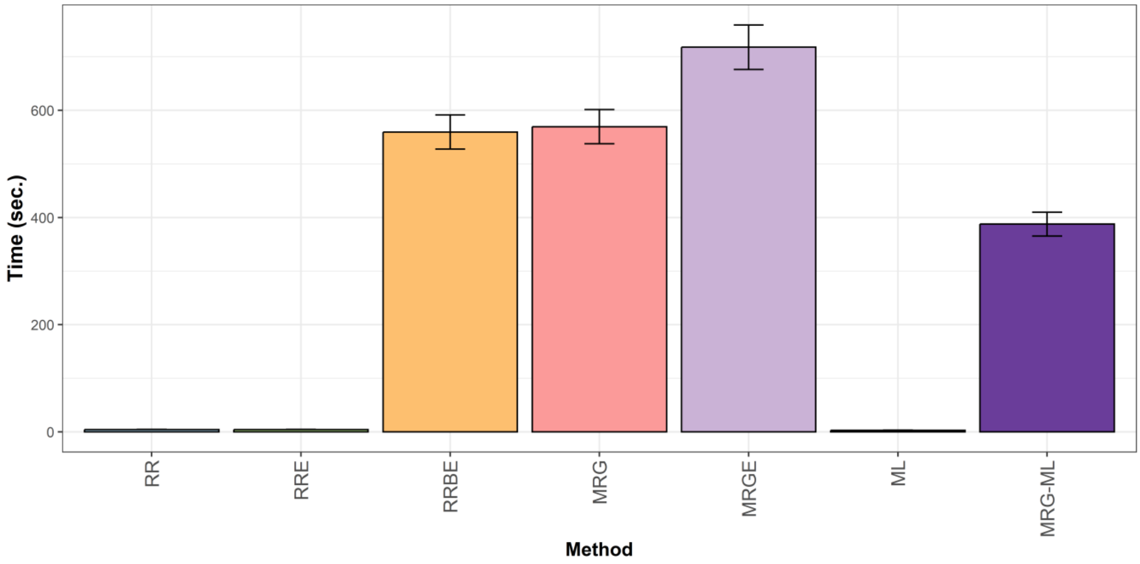

Figure S23. Bar graph of the average execution time of method (Time) in seconds for the "Wheat_5" dataset. The seven methods issued in this study are compared (RR, RRE, RRBE, MRG, MRGE, ML and MRG-ML).

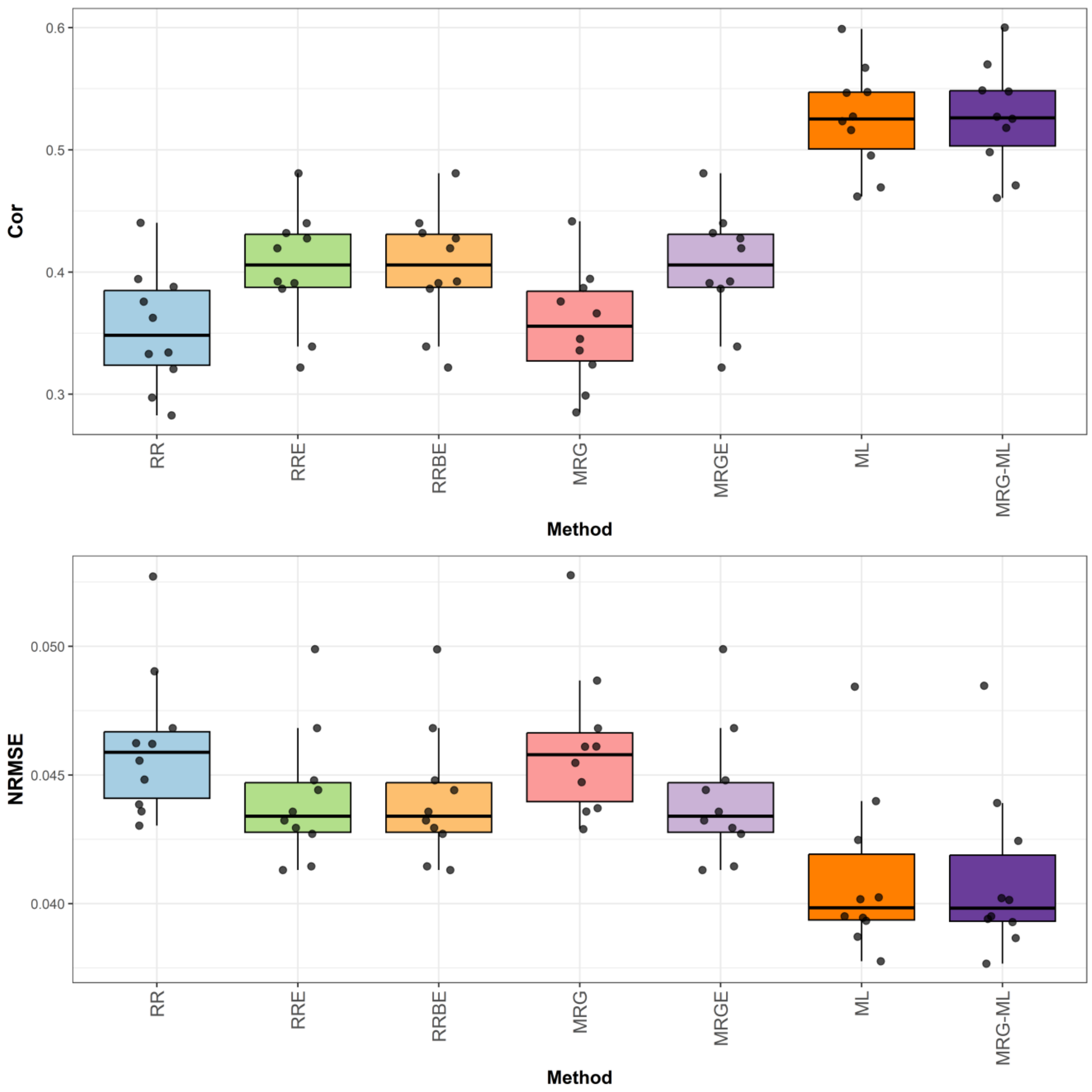

Figure S24. Box plots of Pearson's correlation (Cor) and normalized root mean square error (NRMSE) for the "Wheat_6" dataset are presented in the top and bottom graphs respectively. Each black dot represents a data point per fold and trait.

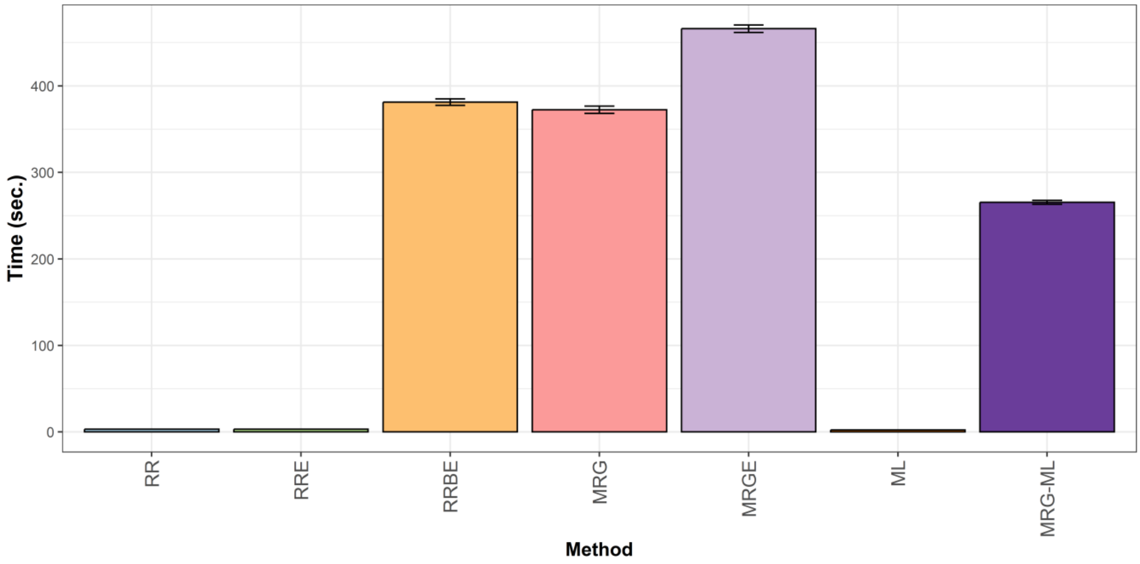

Figure S25. Bar graph of the average execution time of method (Time) in seconds for the “Wheat_6” dataset. The seven methods issued in this study are compared (RR, RRE, RRBE, MRG, MRGE, ML and MRG-ML).
